# The ParB homologs, Spo0J and Noc, together prevent premature midcell Z ring assembly when the early stages of replication are blocked in *Bacillus subtilis*

**DOI:** 10.1111/mmi.14319

**Published:** 2019-06-11

**Authors:** Isabella V. Hajduk, Riti Mann, Christopher D. A. Rodrigues, Elizabeth J. Harry

**Affiliations:** ^1^ The ithree institute University of Technology Sydney Po Box 123 Broadway NSW 2007 Australia

## Abstract

Precise cell division in coordination with DNA replication and segregation is of utmost importance for all organisms. The earliest stage of cell division is the assembly of a division protein FtsZ into a ring, known as the Z ring, at midcell. What still eludes us, however, is how bacteria precisely position the Z ring at midcell. Work in *B. subtilis* over the last two decades has identified a link between the early stages of DNA replication and cell division. A recent model proposed that the progression of the early stages of DNA replication leads to an increased ability for the Z ring to form at midcell. This model arose through studies examining Z ring position in mutants blocked at different steps of the early stages of DNA replication. Here, we show that this model is unlikely to be correct and the mutants previously studied generate nucleoids with different capacity for blocking midcell Z ring assembly. Importantly, our data suggest that two proteins of the widespread ParB family, Noc and Spo0J are required to prevent Z ring assembly over the bacterial nucleoid and help fine tune the assembly of the Z ring at midcell during the cell cycle.

## Graphical abstract

Division site selection in *B. subtilis* is thought to be regulated by the progression of initiation of DNA replication which ‘potentiates’ midcell for Z ring assembly. We investigate the involvement of chromosome organisation proteins in this model. Our data do not support the ‘potentiation’ model, and instead suggest that the activity of Spo0J and Noc is all that is required to prevent midcell Z rings forming when the early stages of DNA replication are blocked.

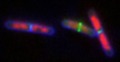

## Introduction

Proper division site selection is essential for bacterial cell propagation and survival. In bacteria such as *Escherichia coli* and *Bacillus subtilis*, cell division is instigated by the polymerisation of the highly conserved tubulin‐like protein, FtsZ, into a cytokinetic Z ring at the centre of the cell, between segregated chromosomes (Haeusser and Margolin, 2016; Hajduk *et al.*, 2016). How Z ring positioning is coupled to other cell cycle events, including DNA replication and segregation remains a significant question.

Two well‐known negative regulators for the assembly of the Z ring at midcell in many rod‐shaped bacteria, including *B. subtilis* and *E. coli*, are the Min system (Min) and nucleoid occlusion. The Min system is composed of several proteins and its primary role is to inhibit Z ring formation at the nucleoid‐free cell poles (Shih and Zheng, 2013; Margolin and Rowlett, 2015). Nucleoid occlusion prevents Z ring formation over the nucleoid until the majority of the chromosomes have been segregated relieving nucleoid occlusion at midcell, allowing the Z ring to form at the cell centre (Wu and Errington, 2004; Bernhardt and de Boer, 2005). In *B. subtilis*, Noc, a homologue of ParB, is a nucleoid occlusion protein that has several DNA‐binding site on the chromosome essential for its activity (Wu *et al.*, 2009). A recent model has postulated that Noc, by forming nucleoprotein complexes on the DNA and simultaneously binding the membrane, creates a region of high molecular crowding at the membrane periphery which physically occludes Z ring assembly over the nucleoid (Adams *et al.*, 2015). Notably, Noc is only essential when DNA replication or segregation is perturbed, to prevent guillotining of the chromosome by the division machinery (Wu and Errington, 2004; Bernhardt and de Boer, 2005).

Interestingly, cells lacking Noc are still capable of preventing the division septum guillotining of the DNA when DNA replication or segregation are perturbed (Bernard *et al.*, 2010; Moriya *et al.*, 2010). This has led to the idea that other as yet unknown Noc‐independent nucleoid occlusion effects are at play in *B. subtilis* (Bernard *et al.*, 2010). Furthermore, while Min and Noc play a role in ensuring proper cell division, neither are required for precise midcell Z ring positioning in *B. subtilis*. Thus, these systems do not dictate where the Z ring forms. Instead, they allow for the efficient utilisation of the midcell site (Rodrigues and Harry, 2012). Other mechanisms must exist to ensure precise midcell assembly of the Z ring in *B. subtilis*. Similar findings have been made in *E. coli* regarding the essentiality of Min and nucleoid occlusion protein SlmA in Z ring positioning (Bernhardt and de Boer, 2005; Bailey *et al.*, 2014). Furthermore, in several other organisms including *Streptococcus pneumoniae, Myxococcusxanthus* and *Streptomyces coelicolor*, the spatial regulation of cell division occurs by unrelated mechanisms that influence Z ring assembly in a positive manner (Traag and van Wezel, 2008; Willemse *et al.*, 2011; Treuner‐Lange *et al.*, 2013; Fleurie *et al.*, 2014; Holečková *et al.*, 2015).

Clues as to what might determine the correct positioning of the Z ring in *B. subtilis*, relates to a putative link between DNA replication and Z ring positioning. Studies over the last two decades have suggested that correct positioning of the division site at midcell in *B. subtilis* is linked to the early stages of DNA replication (Wu *et al.*, 1995; Harry *et al.*, 1999; Regamey *et al.*, 2000; Moriya *et al.*, 2010). Our data in this area led us to propose the Ready‐Set‐Go model (Moriya *et al.*, 2010). In this model, the steps required for the early stages of DNA replication, that is, the initiation phase leading up to complete replisome assembly at the origin of replication and entry into elongation, potentiate the midcell site for Z ring assembly. However, this site only becomes available later in the round of replication, when chromosomes have replicated and segregated, thus relieving the effects of nucleoid occlusion over the midcell site. Importantly, this ‘potentiation’ effect is only seen in the absence of Noc, which led to the Ready‐Set‐Go idea that the midcell division site is ‘set’ early in the round of replication but only ready to ‘go’ later in the cell cycle when chromosomes have been replicated and segregated and Noc is cleared from midcell. How this ‘potentiation’ effect could occur at a molecular level remains unclear.

Interestingly, depending how we inhibited DNA initiation, we observed different nucleoid morphologies that correlated with the ability of a Z ring to form at midcell or not. Midcell Z rings most commonly formed over unreplicated bilobed nucleoids (in which Z rings formed between the bilobes) rather than single‐lobed nucleoids (Moriya *et al.*, 2010). Thus, an alternative possibility to the ‘potentiation’ effect was that inhibition of the early stages of DNA replication impacts an aspect of chromosome organisation that affects nucleoid morphology and therefore midcell Z ring assembly. Although it has been shown that affecting chromosome organisation in *B. subtilis* can impact nucleoid morphology and consequently Z ring positioning, the molecular players that help drive this effect and how they do so is less clear.

Two important players in chromosome organisation/segregation are Soj and Spo0J. These proteins are homologues of the highly conserved *parABS* system and are present in a number of organisms (Livny *et al.*, 2007). Revealed roles for Soj and Spo0J include chromosome organisation and segregation, initiation of DNA replication, and sporulation. One of the first observations implicating Spo0J in chromosome segregation, was that its absence leads to a 100‐fold increase in the formation of anucleate cells in *B. subtilis* (from 0.01% to 1–2% anucleate cells in the population) (Ireton *et al.*, 1994). Anucleate cells are believed to arise from nucleoid occlusion preventing Z ring formation from occurring over the unsegregated DNA resulting from the absence of Spo0J. Further studies revealed that the absence of Spo0J results in chromosome decondensation in about 20% of cells (Autret *et al.*, 2001). Soj, on the contrary, does not have notable effect on chromosome segregation like Spo0J, instead studies have revealed a role for Soj (ParA), a Walker‐type ATPase, as a direct regulator of the DNA replication initiation protein, DnaA (Murray and Errington, 2008).

Chromosome capture (HiC) (Marbouty *et al.*, 2015; Wang *et al.*, 2015) studies suggest that Spo0J plays an important role in chromosome organisation by mediating both short and long‐range interactions between chromosomal arms, forming Spo0J‐nucleoprotein complexes (Murray *et al.*, 2006; Breier and Grossman, 2007; Graham *et al.*, 2014). These complexes then appear to act as the primary beacon for the SMC (Structural Maintenance of Chromosome) condensin complex, along with origin‐proximal ribosomal DNA (Gruber and Errington, 2009; Yano and Niki, 2017), bringing about origin resolution and segregation (Wang *et al.*, 2014a).

In this study, we investigated whether Soj and/or Spo0J have a role in coordinating the early stages of DNA replication with midcell Z ring assembly in the model organism *B. subtilis*. We show that Spo0J, but not Soj, is required to prevent midcell Z ring positioning when the early stages of DNA replication are blocked. Surprisingly, we found that Spo0J and Noc act together to prevent Z ring assembly over unreplicated DNA, as a *spo0J noc* double mutant allows for wild‐type levels of midcell Z ring assembly, regardless of the block imposed at the early stages of DNA replication. This important result suggests that division‐site selection in *B. subtilis* is not potentiated by the progression of the early stages of DNA replication as proposed in the Ready‐Set‐Go model. Instead, Spo0J together with Noc, prevent all Z rings from forming at midcell when the early stages of DNA replication are blocked. Furthermore, our data suggest that the negative effect Spo0J mediates on Z ring positioning is due to a combination of Spo0J activity itself and its role in chromosome organisation through recruitment of SMC. Finally, we show that Noc and Spo0J are both required to prevent premature midcell Z ring assembly in cells undergoing active DNA replication.

## Results

### Spo0J is required to prevent midcell Z ring assembly when the early stages of DNA replication are blocked

Previously in the course of our analysis of the relationship between Z ring positioning and the early stages of DNA replication, we observed that, generally, midcell Z ring positioning correlated with an unreplicated nucleoid in the form of bilobed morphology and acentral Z ring positioning correlated with an unreplicated nucleoid in the form of single‐lobed morphology (Moriya *et al.*, 2010). This apparent correlation between nucleoid morphology and the ability to assemble a Z ring at midcell, hinted that the link between DNA replication initiation and Z ring positioning could relate to an aspect of chromosome organisation that arises as DNA replication attempts to initiate. Two well studied proteins, Spo0J and Soj, in *B. subtilis* have been shown to have roles in DNA replication and chromosome organisation (Ogura *et al.*, 2003). Specifically, Spo0J has been implicated in the organisation of the chromosome (Graham *et al.*, 2014), while both proteins have been shown to be involved in the regulation of DNA replication initiation (Murray and Errington, 2008; Scholefield *et al.*, 2011). Thus, we considered the possibility that the activities of either Spo0J or Soj, or both, could be required to generate a specific chromosome organisation that influences Z ring positioning.

As a first test to this possibility, we examined Z ring positioning in a *soj‐spo0J* double mutant when DNA replication initiation is inhibited using the temperature‐sensitive *dnaB* mutation, *dna‐1*. We used the spore outgrowth system (unless otherwise stated) because in this system, following a shift of *dna‐1* mutant cells to the non‐permissive temperature to block replication initiation, we can examine Z ring positioning as a consequence of that inhibition to very first round of replication, without any complication from previous rounds. Specifically, spores of the *dna‐1* mutant were germinated at 34°C for 20 min and then shifted to the non‐permissive temperature (48°C) to inactivate DnaB. Cells were then harvested at the point at which the first Z ring appears, in cells of similar lengths. Because of this, specific incubation periods vary amongst strains used simply due to the varying germination and outgrowth times (detailed in the Figure legends). Unless otherwise stated, Z rings were co‐visualised using a xylose‐inducible *ftsZ‐yfp* fusion, induced minimally (0.02% xylose), integrated at the *amyE* locus (Migocki *et al.*, 2002; Migocki *et al.*, 2004; Peters *et al.*, 2007). However, all Z ring positioning and nucleoid morphology results were also confirmed via immunofluorescence and DAPI staining of fixed cells, respectively.

**Figure 1:**
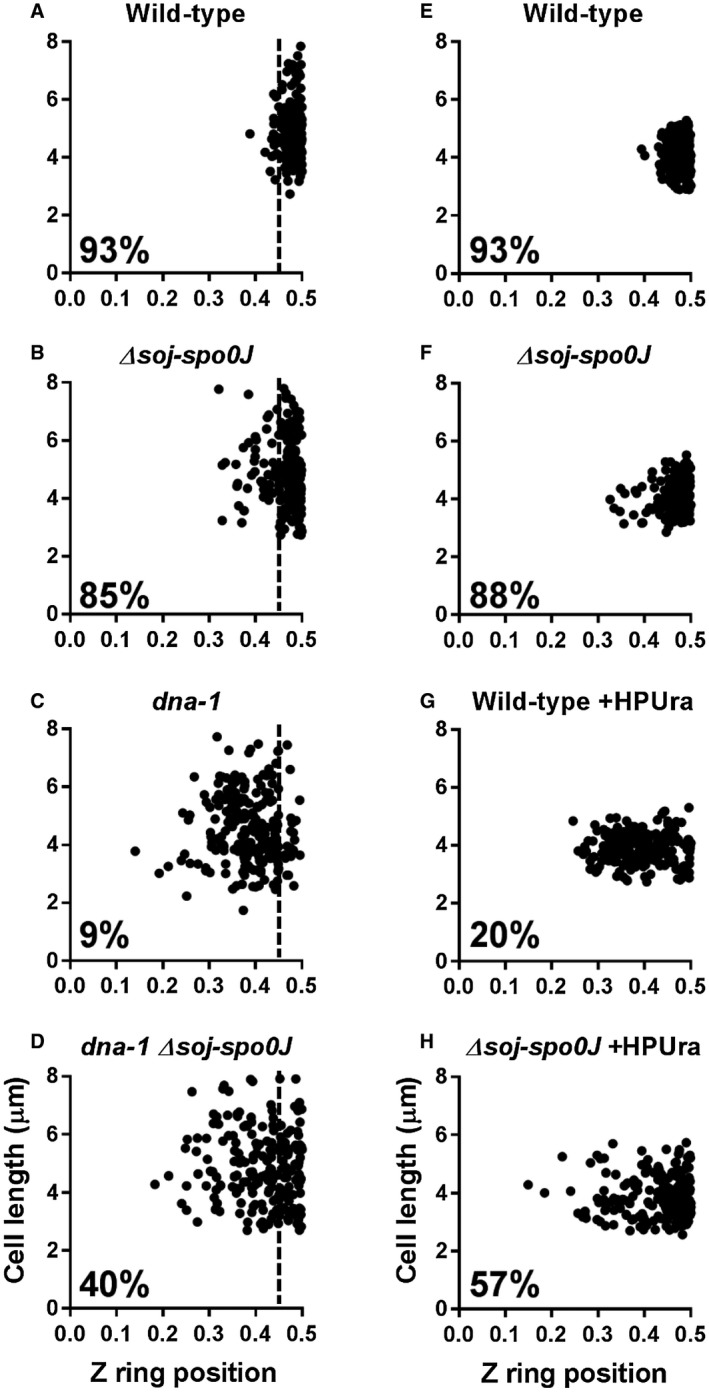
Z ring positioning when the early stages of DNA replication are blocked during spore outgrowth. Z ring positioning was examined in two conditions: in the temperature‐sensitive *dna‐1* background (A–D) and with the addition of the DNA polymerase III inhibitor HPUra (E–H). A–D. Spores were germinated in GMD containing 0.02% xylose (v/v) for 20 min at the permissive temperature (34°C), then shifted to the non‐permissive temperature (48°C) for a further 90 min: (A) wild‐type; SU492, (B) *dna‐1*; SU746, (C) *Δsoj‐spo0J*; SU767and (D) *dna‐1 Δsoj‐spo0J*; SU768. E–H. Spores were germinated in GMD containing 0.02% xylose (v/v), in the absence or addition of HPUra (100 μM) at 34°C: (E) wild‐type; SU492, (F) *Δsoj‐spo0J*; SU767, (G) wild‐type +HPUra, (H) *Δsoj‐spo0J* HPUra). Percentages shown are the frequencies of Z rings occurring at midcell in the range of 0.45 – 0.5 on the x‐axis. The vertical dotted line on graphs A–D mark the 0.45 point on the x‐axis. Data point on the right‐hand side of this vertical dotted line are considered midcell. *n* > 200 for each strain.

Z ring positioning is determined by the distance between the Z ring and the cell pole divided by the cell length, with 0.5 indicating midcell. In wild‐type conditions, most (around 85% or above) Z rings are positioned between 0.45 and 0.5 (Fig. 1A). Consistent with previously published results (Harry *et al.*, 1999; Moriya *et al.*, 2010), in the *dna‐1* mutant at the non‐permissive temperature, Z rings were predominantly acentral, with only 9% of the Z rings being positioned at midcell. This is in contrast to the wild‐type strain under the same conditions, where 93% of the Z rings were positioned at midcell (Fig. 1A). A near‐wild‐type midcell Z ring frequency was observed in *soj‐spo0J* (85%) in otherwise wild‐type cells under these same conditions (Fig. 1B). Surprisingly, however, deletion of *soj‐spo0J* in the *dna‐1* condition resulted in an increase in midcell Z ring frequency from 9% to 40% (Fig. 1D). Similar results were obtained for vegetative cells (Fig. S1). This suggests that either Soj or Spo0J (or both) contribute to the acentral Z ring phenotype of the *dna‐1* mutant at the non‐permissive temperature.

To determine if either *soj* or *spo0J* was responsible for the acentral Z rings observed in the *dna‐1* mutant, individual deletions of these genes were examined in the *dna‐1* mutant background at the non‐permissive temperature. In this case, we resorted to vegetatively growing cells, since single mutants of *spo0J* or *soj* fail to produce spores (Ireton *et al.*, 1994; Quisel and Grossman, 2000). Interestingly, the *dna‐1 spo0J* condition resulted in the same Z ring positioning frequency as that seen in *dna‐1 soj‐spo0J* (40% versus 39% midcell Z rings, respectively), while *dna‐1 soj* showed no difference in midcell Z ring frequency compared to the control *dna‐1* condition (Fig. S2). This suggests that Spo0J, but not Soj, is required for preventing acentral Z ring assembly when initiation of DNA replication is blocked. To facilitate the use of the outgrown spore system, we used the *soj‐spo0J* mutant that is able to form spores for the remainder of this work. However, where possible, all the different mutants and conditions mentioned, except for the HPUra experiments, were also tested during vegetative growth to ensure that the data obtained from the spore outgrowth system was in agreement with the vegetative cell data. Importantly, the *dna‐1* mutant at the permissive temperature showed essentially the same frequency of midcell Z rings as wild type (Fig. S3).

To examine whether Spo0J is also required for acentral Z ring positioning in conditions that block entry into DNA elongation, we used the DNA polymerase III inhibitor, HPUra (6‐para‐hydroxyphenylazo)‐uracil). HPUra blocks DNA replication in *B. subtilis* by binding to DNA polymerase III, preventing its progression along the DNA template (Brown, 1970; Bazill and Gross, 1972). However, when HPUra is added at the beginning of spore germination and outgrowth, DNA replication is blocked at the onset of DNA synthesis. Consistent with previous results, wild type and *soj‐spo0J* mutant spores resulted in 93% and 88% midcell Z rings when no HPUra was added respectively (Fig. 1E and F). Wild‐type spores grown in the presence of HPUra resulted in 20% midcell Z rings; however, the midcell Z ring frequency increased to 57% in the *soj‐spo0J* mutant (Fig. 1G and H). Thus, as with the *dna‐1* mutant, Spo0J contributes to acentral Z ring positioning when the early stages of DNA replication are blocked via the addition of HPUra. Collectively, these results suggest that Spo0J is required to prevent midcell Z ring assembly when the early stages of DNA replication are blocked.

### Spo0J helps to maintain a specific nucleoid morphology that prevents midcell Z ring assembly when the early stages of DNA replication are blocked

**Figure 2:**
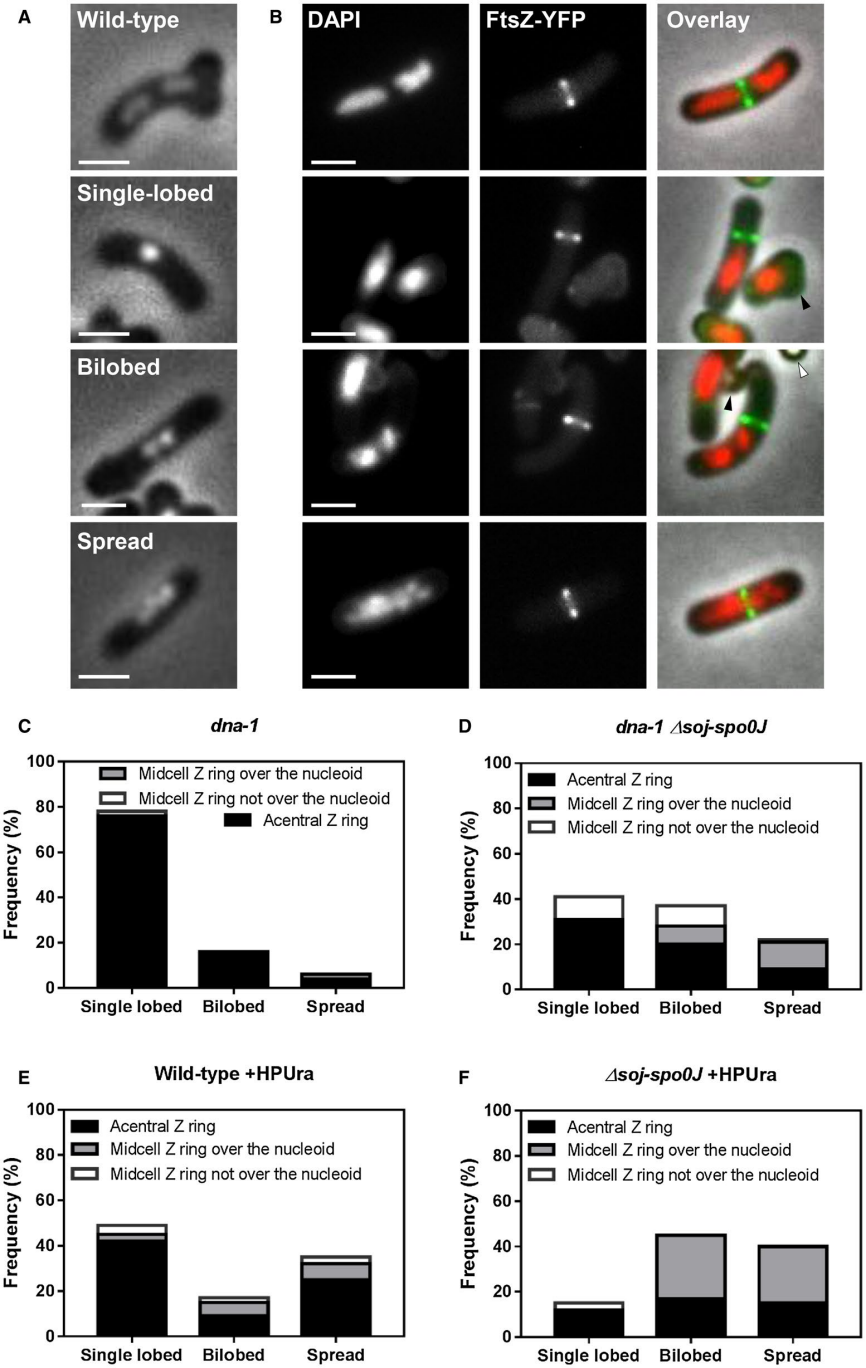
Dual analysis of nucleoid morphologies and Z rings positions when the early stages of DNA replication are blocked in the absence of *soj‐spo0J*. A. Different nucleoid morphologies observed including a wild‐type cell with replication occurring normally, and when initiation of DNA replication is blocked: (top to bottom) wild‐type, single‐lobed, bilobed and spread nucleoid. B. Representative images of wild‐type cells (top image) and predominant combinations Z ring positions and nucleoid types quantified when the early stages of DNA are blocked: acentral Z ring adjacent to a single‐lobed nucleoid, central Z ring forming over a spread nucleoid, acentral Z ring adjacent to spread nucleoid and acentral Z ring forming over a spread nucleoid. Representative images above show in each column: DAPI (0.4 µg ml^−1^; left); FtsZ‐YFP (middle); and an overlay of the three preceding images (right). Images also show ungerminated spores (white arrows) and remnant spore coats (black arrows). Scale bar represents 2 μm. C–F. Histogram representation of Z ring position relative to different nucleoid type in (C) *dna‐1;* SU764, (D) *dna‐1 Δsoj‐spo0J;* SU768, (E) Wild‐type; SU492 +HPUra and (F) *Δsoj‐spo0J*; SU767 +HPUra*.* Spore samples were germinated and grown at either (C–D) the permissive temperature for 20 min and then shifted to the non‐permissive for a further 90 min; or (E–F) the permissive temperature for 120 min with media supplemented with HPUra (100 μM). The height of each bar represents the frequency of each nucleoid type, showing the proportion of acentral Z rings (black), midcell Z rings over the nucleoid (grey) and midcell Z rings not over the nucleoid (white). *n* > 200 for each strain.

Since Spo0J is involved in chromosome organisation (Sullivan *et al.*, 2009; Graham *et al.*, 2014; Chen *et al.*, 2015), we questioned whether the increase in centrally located Z rings observed in the *soj‐spo0J dna‐1* mutant and in the *soj‐spo0J* +HPUra situation were due to changes in nucleoid morphologies. In other words, could Spo0J be acting by maintaining a specific nucleoid morphology during the early stages of DNA replication that inhibits the assembly of midcell Z rings? To examine this, we visualised the Z ring and the nucleoid in the same cells using FtsZ‐YFP and DAPI (4′6‐diamidino‐2‐phenylindole) respectively. Three different nucleoid morphologies were observed (see Fig. 2A): cells with a single region of DNA were denoted as ‘single‐lobed’; cells with two adjacent regions of DNA were termed ‘bilobed’; and cells with DNA that had a spread appearance through a large portion of the cell were denoted ‘spread’. We also determined the frequency of cells containing a particular nucleoid type associated with either an acentral or midcell Z ring (Fig. 2C and D). Representative examples of the different combinations between nucleoid type and Z ring position are shown in Fig 2B (see Fig. S4 for larger fields of cells).

Consistent with previous reports (Harry *et al.*, 1999; Moriya *et al.*, 2010), the predominant nucleoid morphology in the *dna‐1* strain at the non‐permissive temperature was the single‐lobed nucleoid (Fig. 2C), at a frequency of 78%. Bilobed and spread nucleoid represented a minority of the population (16% and 6% respectively). In contrast, combining the *soj‐spo0J* mutant with *dna‐1* at the non‐permissive temperature resulted in a two‐fold increase in frequency of bilobed nucleoids (37% versus 16% respectively), as well as a significant increase in the frequency of spread nucleoids (from 6% to 22%). Importantly, consistent with the idea that Soj does not affect chromosome organisation (Ireton *et al.*, 1994; Lee and Grossman, 2006), the changes in nucleoid morphology we observed in the *dna‐1 soj‐spo0J* mutant are due to *spo0J* and not *soj* (Fig. S2). Similar changes in nucleoid morphology where also observed when HPUra was added to the *soj‐spo0J* mutant (Fig. 2F). In otherwise wild‐type cells grown in the presence of HPUra, the predominant nucleoid morphology was single‐lobed nucleoids (48%; Fig. 2E). In contrast, when HPUra was added to *soj‐spo0J* mutant cells, fewer single‐lobed nucleoids were observed (15%; Fig. 2F). Instead nucleoids were predominantly bilobed or spread (46% and 39%, respectively). This data suggest that when the early stages of DNA replication are inhibited, Spo0J is required to maintain the nucleoid in a condensed morphology.

We next examined how the change in nucleoid morphologies in the *soj‐spo0J* strains correlated with Z ring positioning. Consistent with previous results (Moriya *et al.*, 2010), in the *dna‐1* mutant at the non‐permissive temperature acentral Z rings were observed in cells containing both single‐lobed and bilobed nucleoids. The few midcell Z rings observed were in cells containing single‐lobed nucleoids that appeared to have moved away from the cell centre or in cells with spread nucleoids (Fig. 2C). In the *dna‐1 soj‐spo0J* mutant at the non‐permissive temperature, we observed that the increase in bilobed and spread nucleoids correlated with an increase in the number of cells containing midcell Z rings over these nucleoids (8% over bilobed and 12% over spread nucleoids; Fig. 2D). Midcell Z rings were also observed when either a single‐lobed or bilobed nucleoid moved away from the cell centre (10% in off‐centre single‐lobed and 9% in off‐centre bilobed nucleoids). Acentral Z rings were predominantly observed in cells containing a single‐lobed nucleoid (30%), but also in cells containing bilobed or spread nucleoids (20% and 9% respectively). Thus, the increase in midcell Z ring assembly observed in the *dna‐1 soj‐spo0J* mutant at the non‐permissive temperature correlates with either the nucleoid adopting a less‐condensed morphology (spread and bilobed) or with the nucleoid moving away from the midcell position.

A similar trend regarding midcell Z ring positioning and nucleoid morphology was observed when HPUra was added to *soj‐spo0J* mutant. In the wild‐type background, and consistent with previous results (Moriya *et al.*, 2010), the most predominant phenotype observed with HPUra addition was an acentral Z ring, positioned to the side of a centrally positioned single‐lobed nucleoid (43% of all phenotypes observed; Fig. 2E). Of the few Z rings that did form at midcell over the DNA in this condition, the nucleoids were either bilobed or spread (6% and 7%, respectively). In the absence of *soj‐spo0J*, however, almost all Z rings that formed at midcell formed over either a spread or bilobed nucleoid (25% and 28%, respectively; Fig. 2F). Collectively, these results show that the increase in bilobed or spread morphology observed in both the *dna‐1 soj‐spo0J* and +HPUra *soj‐spo0J* mutant conditions correlates with an increase in midcell Z ring formation, with a significant proportion of midcell Z rings assembling over spread or bilobed nucleoids.

Given the dramatic increase in midcell Z rings and changes to nucleoid morphology observed in the *dna‐1 soj‐spo0J* and +HPUra *soj‐spo0J* mutant conditions, we questioned whether the increase in midcell Z ring frequency observed is attributed to a relief in the block to initiation of DNA replication. Therefore, to rule out the possibility that some active replication was occurring in these mutant conditions and Z rings could more readily form at the centre, we used a flow‐cytometry approach using the DNA dye SYTO16, as used previously (Okumura *et al.*, 2012). We found that the DNA content in the *soj‐spo0J* mutants is the same as the Soj‐Spo0J+ conditions (Fig. S5). Thus, the increase in midcell Z rings in the *soj‐spo0J* mutant strains inhibited for DNA replication initiation is not due to any relief of this inhibition, which would give rise to DNA synthesis.

Collectively, these results suggest that the absence of Spo0J results in changes to chromosome organisation that favour midcell Z ring assembly despite the inhibition of the early stages of DNA replication.

### Both Spo0J and Noc are required to completely prevent midcell Z ring assembly when the early stages of DNA replication are blocked

Previously we had shown that, like Spo0J, Noc was required to prevent midcell Z ring assembly in the *dna‐1* mutant and the +HPUra condition (Moriya *et al.*, 2010). In fact, the increase in the frequency of midcell Z rings in a *dna‐1 noc* mutant and +HPUra *noc* mutant is very similar to what we observed under these conditions in the absence of *soj‐spo0J.* Noc has been shown to bind the chromosome at specific DNA sequences and this binding is required for its activity as a nucleoid occlusion factor (Wu *et al.*, 2009). Thus, we questioned if the increase in midcell Z ring positioning observed in the absence of *soj‐spo0J* could be explained by pleotropic effects on Noc activity. In other words, could the changes in nucleoid morphology in the absence of *soj‐spo0J* impact Noc localisation such that Noc‐associated nucleoid occlusion was partly relieved? If so, then we might see Noc no longer co‐localising with the DNA as commonly seen in wild‐type cells (Wu *et al.*, 2009; Adams *et al.*, 2015). To test this, we first examined Noc localisation using the previously reported Noc‐YFP fusion (Wu *et al.*, 2009). In wild‐type replicating cells, Noc‐YFP localises around the nucleoid in the form of puncta near the membrane periphery (Adams *et al.*, 2015; Fig. S6A). A similar localisation of Noc‐YFP was observed in a *soj‐spo0J* mutant (Fig. S6C). In the *dna‐1* mutant at the non‐permissive, Noc‐YFP localised in a similar fashion as observed in wild‐type cells replicating normally, with puncta accumulating in the periphery of the membrane surrounding the unreplicated nucleoid (Fig. S6B). An identical pattern of Noc‐YFP localisation was observed in the *dna‐1 soj‐spo0J* mutant at the non‐permissive temperature (Fig. S6D). These results suggest that in the *soj‐spo0J* mutant when initiation of DNA replication is blocked, Noc localisation, and likely its activity, is generally not affected despite the observed changes in nucleoid morphology. That is, although the nucleoid morphologies are different, Noc still forms a pattern over the nucleoid that is consistent with what is observed in wild‐type cells.

**Figure 3:**
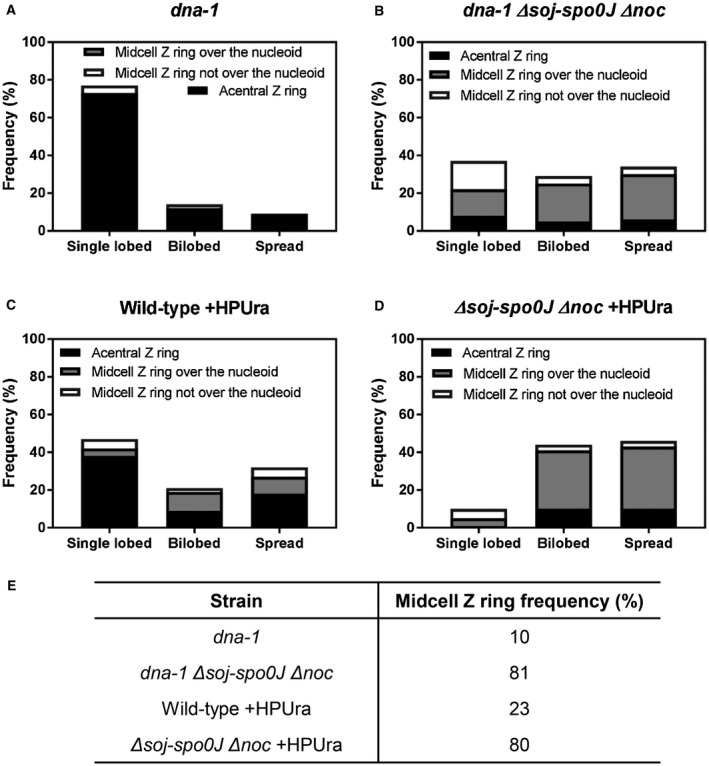
Dual analysis of nucleoid morphologies and Z rings positions when the early stages of DNA replication are blocked in the absence of both *soj‐spo0J* and *noc*. A–D. Histogram representation of Z ring position relative to different nucleoid type in (A) *dna‐1;* SU764, (B) *dna‐1 Δsoj‐spo0J Δnoc;* SU836 (C) Wild‐type; SU492 +HPUra and (D) *Δsoj‐spo0J Δnoc*; SU835 +HPUra*.* E. Midcell Z ring frequencies in the two different replication blocks. *n* > 200 for each strain.

If Noc is still active in a s*oj‐spo0J* mutant, then combining a *noc* mutant with a *soj‐spo0J* mutant, when the early stages of DNA replication are inhibited, should result in a further increase in the number of midcell Z rings to that observed in the separate *soj‐spo0J* and *noc* mutants. To test this, we examined Z ring positioning, and its relationship to nucleoid morphology, in a *noc soj‐spo0J* mutant in both the *dna‐1* mutant at the non‐permissive temperature and when HPUra is added (Fig. 3). Remarkably, for both situations, we observed that virtually all Z rings could assemble at midcell when all three proteins, Noc, Soj and Spo0J are absent. The combined deletion of *soj*, s*po0J* and *noc* essentially completely restored Z ring positioning to wild‐type levels in both the *dna‐1* and +HPUra conditions, 81% and 80% midcell Z rings respectively (compared to wild‐type, ~85% midcell Z rings; Fig. 3E). Again, the differences in Z ring positioning between *noc soj‐spo0J* and *soj‐spo0J* mutants are due to Spo0J and not Soj, as observed in the *dna‐1* mutant (Fig. S8), further highlighting the role of Spo0J specifically in influencing Z ring positioning when DNA replication initiation is blocked. Importantly, complementation of *spo0J* at an ectopic locus, both in *dna‐1 noc soj‐spo0J* and *dna‐1 noc spo0J* mutant strains, as well as their *noc+ *counterpart strains, at the non‐permissive temperature, resulted in similar nucleoid morphologies and very few midcell Z rings (Fig. S9), like that seen in the matching *dna‐1* strains that were otherwise wild‐type for Spo0J (Fig. 1C). This suggests that it is the absence of *spo0J* that is responsible for the increase in midcell Z rings and changes to nucleoid morphology in the *noc soj‐spo0J* deletion strains. In addition, we did not observe significant changes to nucleoid morphology in the *noc soj‐spo0J* triple mutant relative to the *soj‐spo0J* double mutant, both in the *dna‐1* mutant at the non‐permissive temperature and +HPUra condition (Fig. S7), indicating that Noc does not affect the gross morphology of the nucleoid when the early stages of DNA replication are blocked.

Collectively, these data demonstrate that the increase in midcell Z rings observed in the absence of Spo0J is not due to changes to Noc activity. Furthermore, these data support the idea that the activity of both Spo0J and Noc is required to prevent midcell Z ring assembly when the early stages of DNA replication are blocked. Importantly, these data suggest that progression through the early stages of DNA replication does not potentiate midcell for Z ring to assembly, as postulated by the Ready‐Set‐Go model (Moriya *et al.*, 2010), as Z rings are able to form at midcell to wild‐type levels in the absence of *spo0J* and *noc* when the early stages of DNA replication are blocked.

### Spo0J maintains the chromosomal arms in close proximity and contributes to nucleoid compaction when the early stages of DNA replication are blocked

Our data thus far are consistent with the idea that some aspect of chromosome organisation controlled by Spo0J, in addition to the activity of Noc, prevent Z rings from forming at midcell when the early stages of DNA replication are blocked. Previous data have shown that Spo0J mediates a compact chromosome organisation, specifically by keeping the left and right arms of the chromosome in close proximity during DNA replication (Wang *et al.*, 2014a). Therefore, to better understand how Spo0J‐mediated chromosome organisation acts to prevent Z rings from forming at midcell when the early stages of DNA replication are blocked, we sought to investigate the general organisation of the unreplicated chromosome in *dna‐1* cells at the non‐permissive temperature in the presence and absence of *soj‐spo0J* and examine how this correlates with Z ring positioning. To do this, we compared the distance between the left and right arms of the chromosome in conditions of acentral and midcell Z ring assembly. Furthermore, by simultaneously visualising the nucleoid, we would also be able to examine how the different nucleoid morphologies, single‐lobed, bilobed and spread, correlate with the arm distance in terms of general chromosome organisation. To this end, we took advantage of the lacO/lacI‐CFP and tetO/tetR‐YFP short array system to label the −87° and +87° positions on the chromosome, which correspond to the left and right arm of the *B. subtilis* chromosome respectively (Wang *et al.*, 2014a).

**Figure 4:**
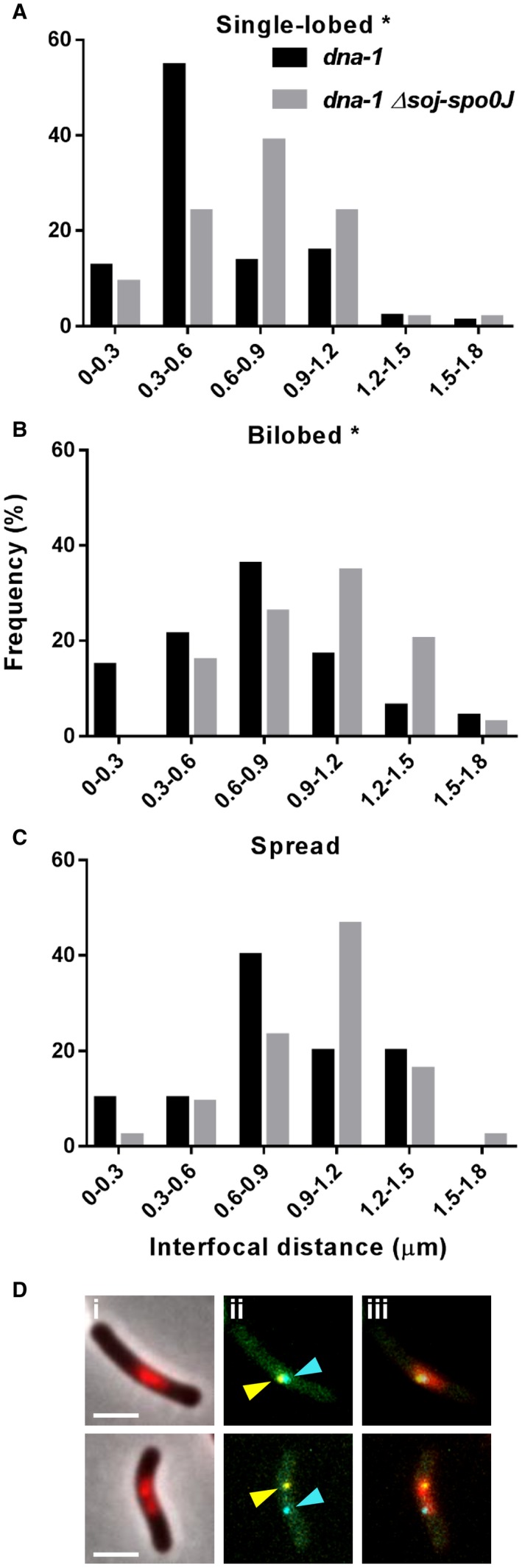
Left and right arm localisation when initiation of DNA replication is blocked via the temperature‐sensitive *dna‐1* mutant. Distances between the +87° and −87° arm were examined in outgrown spores of *dna‐1* (SU895) and *dna‐1 Δsoj‐spo0J* (SU897). Graphs show distance between the +87° and −87° foci in the three different nucleoid morphologies: (A) single‐lobed, (B) bilobed, and (C) spread. Asterisks indicate statistically significant (Kolmogorov‐Smirnov test) differences in focal distances between mutants at *P* < 0.05, as determined in from two replicate experiments. D. Representative images of +87° and −87° arm foci localisation including: (i) phase‐contrast and DAPI overlay, (ii) +87° and −87° foci overlay, and (iii) +87° and −87° foci overlaid with DAPI. Arrows highlight the positions of the +87° (cyan arrows) and −87° (yellow arrows) foci on each arm. Scale bar represents 2 μm. *n* > 100 for each strain.

In the *dna‐1* mutant, for the single‐lobed nucleoids the left and right arms were in close proximity, with an interfocal distance (distance between a LacI‐CFP and TetR‐YFP foci) of 0.6 ± 0.3 μm (Fig. 4A). As for bilobed and spread nucleoids, the interfocal distance was larger (both 0.8 μm; ± 0.3 and ± 0.4 respectively). Interestingly, in the *dna‐1 soj‐spo0J* mutant, we found that the right and left arm were further apart, with the single, bilobed and spread nucleoids, averaging an interfocal distance of 0.8 ± 0.4, 1.0 ± 0.3 and 1.2 ± 0.3 μm respectively. Furthermore, consistent with previous results in *B. subtilis* (Wang *et al.*, 2014a) using other temperature‐sensitive alleles of *dnaB*, we observed that in all of the bilobed nucleoids observed in the *dna‐1 soj‐spo0J* mutant, each lobe of the bilobed contained either a LacI‐CFP or TetR‐YFP, suggesting that each lobe of the bilobed nucleoid corresponds to a separate arm of the chromosome (Fig. 4B). This suggests that the absence of Spo0J results in an increase in the distance between the left and right arm of the chromosome, which likely underlies the dramatic changes in nucleoid morphology we observe from a predominantly single‐lobed in the *dna‐1* mutant to a more bilobed and spread morphology in the *dna‐1 soj‐spo0J* mutant. Furthermore, this data further supports the correlation between changes to chromosome organisation and midcell Z ring assembly. Collectively, our data are consistent with the idea that Spo0J contributes to keeping the left and right chromosomal arm close together, resulting in a more compact nucleoid that actively blocks Z rings from assembly over the DNA.

### Nucleoid compaction, Spo0J and Noc are all that are required to prevent midcell Z rings when the early stages of DNA replication are blocked

**Figure 5:**
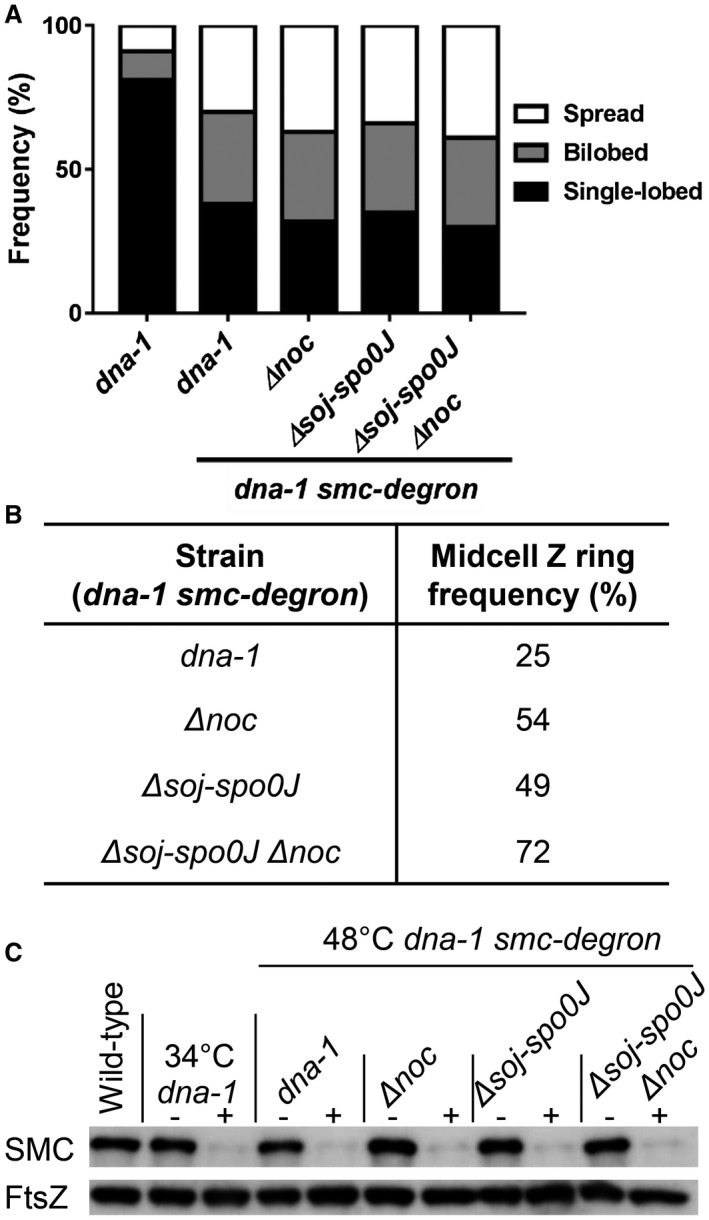
Z ring positioning and nucleoid morphology analysis when SMC is degraded. A. Nucleoid morphologies and B. midcell Z ring frequencies when SMC is degraded in outgrown temperature‐sensitive *dna‐1* mutant spores via the addition of xylose (1% v/v) 15 min prior to shifting the cells to the non‐permissive temperature. C. Immunoblot analysis of SMC and a loading control (FtsZ) in wild‐type (SU5) and the *smc‐ssrA* degradation strains (*dna‐1*; SU851, *Δnoc*; SU875, *Δsoj‐Δspo0J*; SU877, *Δsoj‐Δspo0J Δnoc*; SU879) under conditions in which the adaptor protein is either not induced (−) or induced (+). *n* > 200 for each strain.

Recent work suggests that a major function of Spo0J in chromosome organisation is the recruitment and subsequent loading of the Structural Maintenance of the Chromosome (SMC) condensin complex at origin‐proximal *parS* site (Sullivan *et al.*, 2009; Gruber and Errington, 2009; Graham *et al.*, 2014). Specifically, by recruiting SMC, Spo0J helps to maintain specific interactions between the left and right arm of the chromosomes which allow for efficient chromosome resolution and segregation (Wang *et al.*, 2017). In the absence of either Spo0J or SMC these interactions are lost, resulting in increased distance between the left and right arm of the chromosomes. Since we also observed an increase in separation between the left and right arm of the unreplicated chromosome in the *dna‐1 spo0J* mutant, we considered the possibility that the effects we have observed so far on nucleoid morphology and consequently Z ring positioning in this mutant result from lack of SMC recruitment by Spo0J to the DNA. In others words, SMC is the primary factor that maintains the nucleoid in a confirmation that is more capable of preventing midcell Z ring assembly over unreplicated DNA. If this is the case, then one might expect that a *dna‐1* SMC mutant would phenocopy a *dna‐1 spo0J* mutant with regard to nucleoid morphology and Z ring positioning. To test this possibility, we sought to characterise nucleoid morphology and Z ring positioning in a *dna‐1* SMC mutant at the non‐permissive temperature. Since SMC deletion mutants are unable to grow at temperatures above 30°C, we resorted to the previously characterised SMC‐degradation system (Wang *et al.*, 2014a). The SMC‐degradation system contains the xylose‐inducible *E. coli* adaptor protein SspB which targets SsrA‐tagged proteins (in this case SMC possesses the SsrA tag) to the ClpXP protease leading to degradation of the SsrA‐tagged protein (Wang *et al.*, 2014a). We examined nucleoid morphology and Z ring positioning in the *dna‐1* mutant at the non‐permissive temperature after induction of the SspB adaptor protein and when only approximately 10% of SMC was detected by immunoblot (Fig. 5C).

As predicted, SMC degradation was accompanied by significant changes to nucleoid morphology. We observed an increase in the frequency of bilobed and spread nucleoids to a similar degree as what we observed in the *dna‐1* at the non‐permissive temperature in *soj‐spo0J* background (compare Figs 5B with 2D). Interestingly, while SMC degradation allowed a moderate increase in the number of midcell Z rings (25%, Figs 5B and S9) relative to the *dna‐1*mutant at the non‐permissive temperature where SMC is not degraded (10%), the extent of this increase is less than that observed in a *dna‐1* mutant at the non‐permissive temperature in the *soj‐spo0J* background (40%, Fig. 1D). Thus, while SMC degradation results in similar changes to the *dna‐1* mutant nucleoid morphology as the absence of Spo0J, this is not the case for Z ring positioning. This result suggests that Spo0J‐mediated recruitment of SMC determines the compact nucleoid morphology of the *dna‐1* mutant, however, Spo0J appears to play some other role in preventing midcell Z ring assembly when the early stages of DNA replication are blocked.

If Spo0J does indeed play another role in Z ring positioning in addition to mediating SMC recruitment, then a SMC degradation in the *dna‐1 noc* mutant should not phenocopy the Z ring positioning frequency as seen in the *dna‐1 spo0J noc* double mutant. Interestingly, the absence of *noc* in SMC‐degraded cells only resulted in a two‐fold increase in midcell Z rings (from 25% to 54%) relative to SMC degradation alone (25% midcell Z rings; Figs 5B and S10). Comparatively, a *spo0J noc* double mutant results in a complete relief of the midcell site, allowing for wild‐type levels of Z rings to form at midcell (81% midcell Z rings). Thus, even when Noc is absent and the nucleoid is significantly decompacted due to the loss of SMC, the unreplicated DNA can still exert a negative effect on midcell Z ring assembly, and this effect appears to be mediated by Spo0J. Indeed, the degradation of SMC in the *dna‐1 soj‐spo0J noc* mutant at the non‐permissive temperature resulted in almost near wild‐type levels of midcell Z ring assembly (72%, Figs 5B and S10). Additionally, Z ring positioning in the *dna‐1 soj‐spo0J* mutant at the non‐permissive temperature when SMC is degraded resulted in a two‐fold increase in midcell Z rings in the *dna‐1 soj‐spo0J* mutant (49%) relative to the *dna‐1* mutant when SMC is degraded (25% midcell Z rings; Figs 5B and S10). Importantly, neither the deletion of *noc* or *soj‐spo0J*, nor their combination, resulted in further changes to nucleoid morphologies to those already observed as consequence of SMC degradation (Fig. 5A). Our data suggest that Spo0J mediates an additional negative effect on midcell Z ring assembly, over and above its role in SMC recruitment and nucleoid compaction. Collectively, our data demonstrate that the condensation of the DNA by SMC, Spo0J and Noc are all required to prevent Z ring midcell assembly when the early stages of DNA replication are blocked.

### Spo0J and Noc are required to prevent Z rings from assembling too early at new division sites in cells undergoing DNA replication

**Figure 6:**
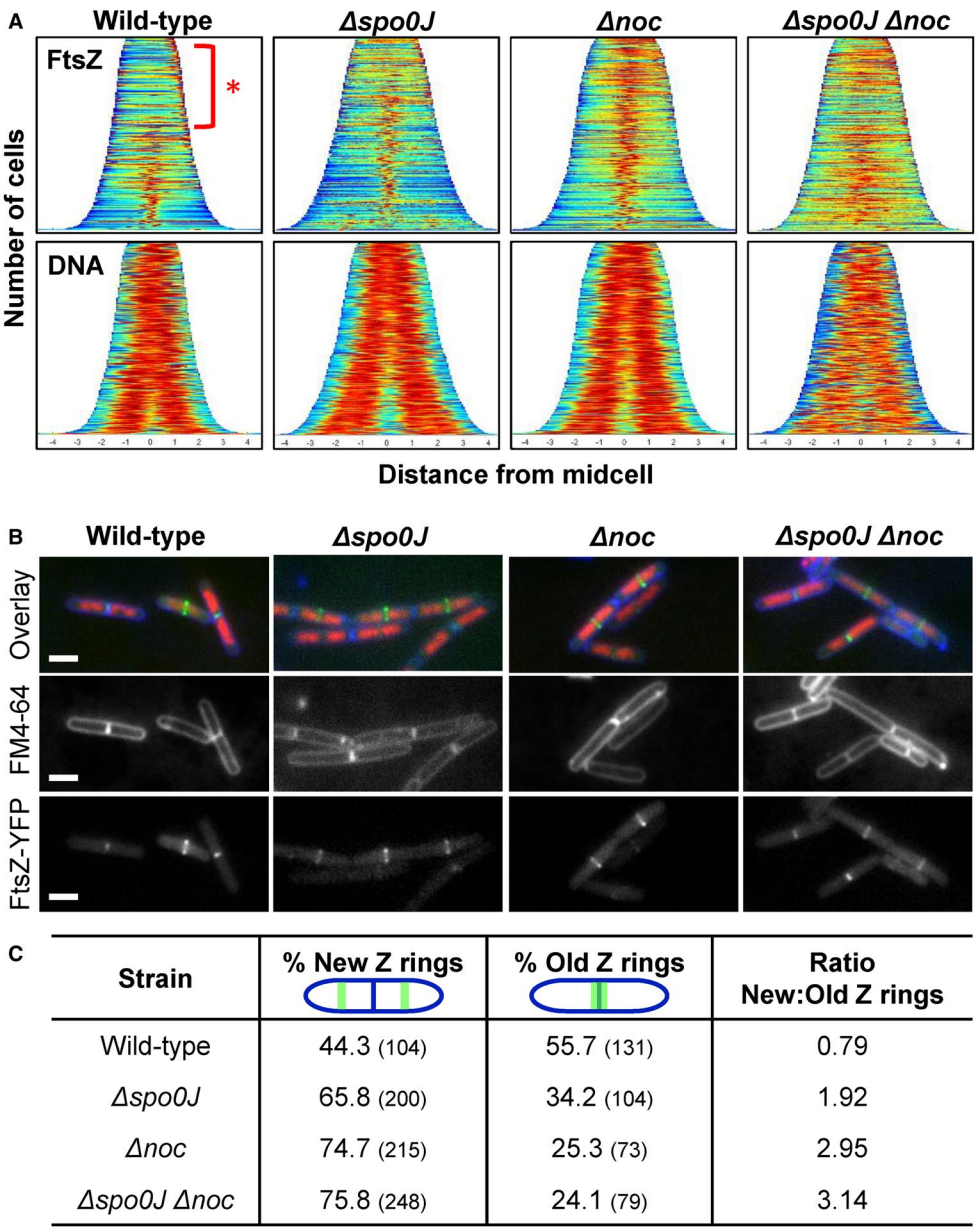
Analysis of Z ring formation in replicating cells in the absence of both *noc* and *spo0J.* Cells were grown in media supplemented with 0.05% xylose to induce FtsZ‐YFP expression, FM4‐64 (3.3 µg ml^−1^) was added 30 min prior to sample collection and DAPI added at the time of collection (0.4 µg ml^−1^). A. Population demographs illustrating localisation of FtsZ (top) and DNA (bottom) in strains (i) wild‐type; SU492, (ii) *Δspo0J*; SU890, (iii) *Δnoc*; SU891, and (iv) *Δspo0J Δnoc*; SU892. *n* > 600 for each strain. B. Representative image and C. Frequencies of new and old Z rings observed in the aforementioned strains. Scale bars represent 2 μm. *n* > 200 for each strain.

Our data suggest that Spo0J and Noc block Z ring assembly at midcell when the early stages of DNA replication are blocked. However, it begs the question of what effect Spo0J and Noc have when the early stages of DNA replication when DNA replication is able to proceed normally. For example, if Spo0J and Noc are indeed preventing Z rings from assembling at midcell too early on in the replication cycle, in normally replicating cells, we may expect to see earlier accumulation of FtsZ at the future (one and three‐quarter) division sites in the cell, prior to proper DNA replication and segregation. Therefore, to further understand the function of Noc and Spo0J, we analysed Z ring assembly in relation to DNA replication and segregation in a large number of cells of the wild‐type, *noc* and *spo0J* mutants and *noc spo0J* double mutants (Fig. 6). To monitor Z ring assembly, all strains contained a xylose‐inducible *ftsZ‐yfp* fusion, induced minimally (0.05% xylose), integrated at the *amyE* locus. Using the FM‐464 dye to stain the membranes and define divided cells, population demographs were generated by plotting FtsZ‐YFP and DAPI fluorescence signal intensity (Fig. 6A and B). In each demograph, the fluorescence signal of each cell within the population analysed was normalised, aligned and ordered according to cell length (increasing from top‐to‐bottom); thus, each demograph provides an overview of the cell cycle based on cell age. In wild‐type cells, FtsZ‐YFP signal intensity started to accumulate at midcell slightly before the DAPI signal could be observed as two different stained regions of the nucleoid within the cell. This is consistent with the idea that FtsZ starts to accumulate at midcell, prior to complete chromosome segregation (Wu *et al.*, 1995). In the *noc* and *spo0J* single mutant, the demographs indicate that the DAPI signal is observed as two separate signals earlier in the cell cycle, compared to what is observed in wild type. This suggests that chromosome segregation occurs slightly earlier in the cell cycle of these mutants. This observation is not so unexpected for the *spo0J* mutant, since Spo0J is required for chromosomal interarm interactions, and loss of these interactions could potentially result in separation of the replicated DNA earlier in the cell cycle. In the case of the* noc* mutant, this observation could reflect a role for Noc in chromosome organisation that only becomes significant by examining a large number of cells (see Discussion). In either mutant, theFtsZ‐YFP signal accumulated at the midcell position at an even earlier stage in the cell cycle, compared to the wild type (Fig. 6A). A similar trend regarding the FtsZ‐YFP signal was observed for the *noc spo0J* double mutant. Collectively, this data does suggest that Noc, Spo0J or both are required to prevent FtsZ accumulation at midcell at an earlier stage of the cell cycle.

Interestingly, we also noticed that in the wild‐type strain many of the cells contained FtsZ‐YFP signal at the older division site (i.e. the nascent pole of cells that have divided but have yet to separate; Fig. 6A – red asterisk), and this signal was less prominent in the *noc* and *spo0J* single mutants or *noc spo0J* double mutant. This suggests that in wild‐type cells, FtsZ‐YFP remains at the older division site for a longer period of time and only once the cells start to segregate their chromosomes, does FtsZ‐YFP start to accumulate at the newer division site. In contrast, in *noc* and *spo0J* mutants, as well as the *noc spo0J* double mutant, FtsZ‐YFP appears to spend less time at the older division site and instead accumulates at the future division site in a larger fraction of cells. To confirm if this was the case, we examined images of these cells and categorised them based on whether the Z ring localised at the old division site, or new division site (Fig. 6B and C). In wild‐type cells, 44% contained Z rings at new division sites, and 56% contained Z rings at older division sites that coincided with already‐formed division septa (new cell poles or divided cells), as judged by membrane staining, with a ratio of new‐to‐old Z rings of 0.8. Interestingly, this ratio increased to 1.9 in the *spo0J* mutant, 2.9 in the *noc* mutant and 3.1 in the *noc spo0J* double mutant. This indicates that cells lacking *spo0J* and *noc* have a higher propensity to accumulate Z rings at new division sites, earlier than that seen in wild‐type cells. Collectively, these results suggest that cells replicating their DNA normally, Spo0J and Noc contribute to fine‐tuning the timing of FtsZ accumulation at the division site. This is consistent with our earlier findings that Spo0J and Noc are required for blocking midcell Z ring assembly when the early stages of DNA replication are blocked.

### Spo0J and Noc are both required to prevent guillotining of the DNA by the division septum

**Figure 7:**
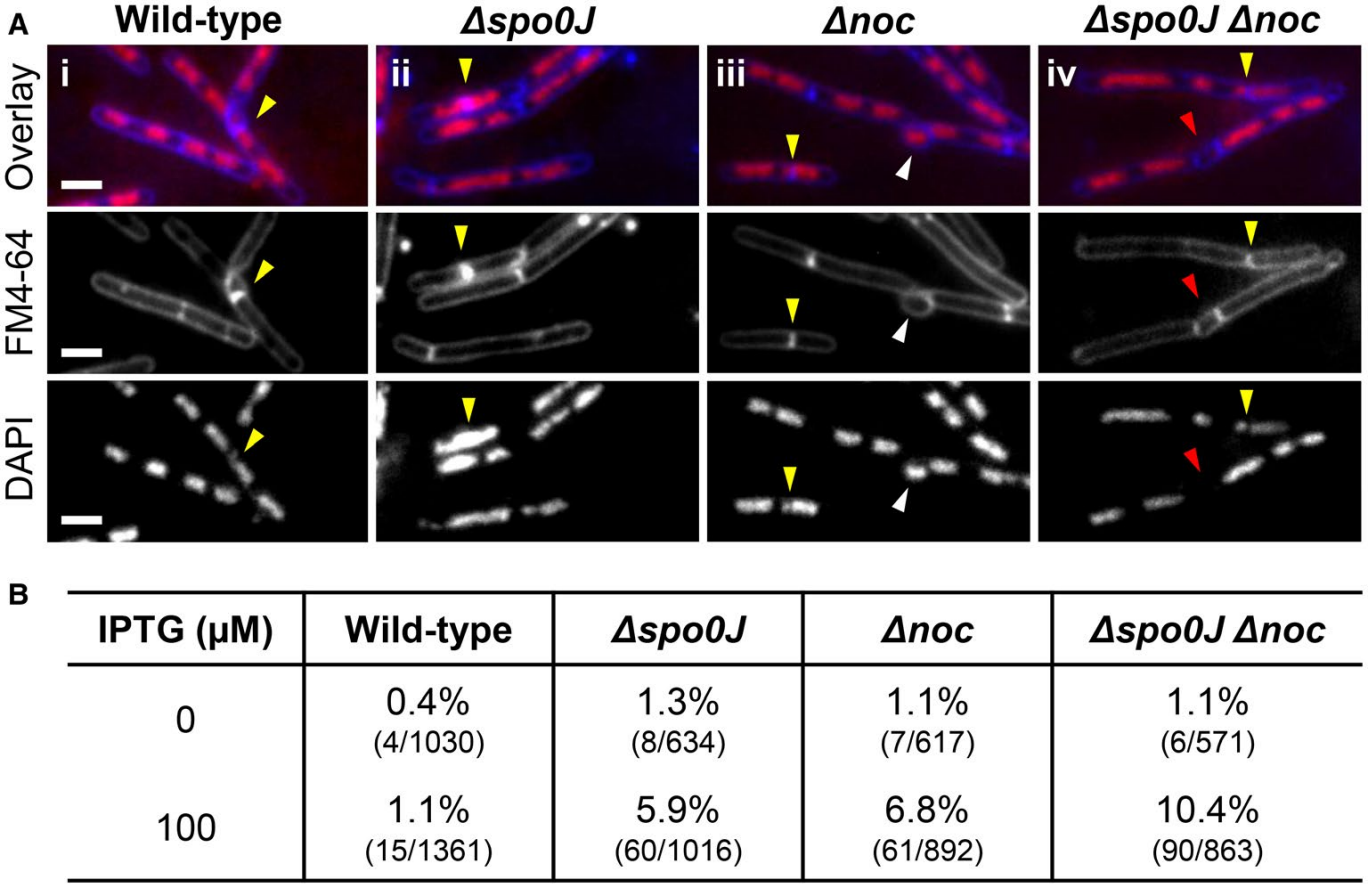
Guillotining of the DNA by the division septum in the absence of both *noc* and *spo0J.* A. Representative examples of cells where the division septum guillotined the DNA (yellow arrows) and B. Frequencies of septum guillotining of the DNA when FtsZ is overexpressed in (i) wild‐type; SU504, (ii) *Δspo0J*; SU887, (iii) *Δnoc*; SU888, and (iv) *Δspo0J Δnoc*; SU889. Cells were grown to mid‐exponential phase, diluted down to 0.05 in duplicate and grown for a further hour with one set of duplicates supplemented with 100 µM IPTG. Representative images show in each row: DAPI (0.4 µg ml^−1^; bottom); FM4‐64 (3.3 µg ml^−1^; middle); and an overlay of the two (top). Images also show additional phenotypes including anucleate cells (red arrows) and bulging formations at the division site (white arrows). Scale bars represent 2 μm.

The above data suggest that Spo0J and Noc prevent Z rings from assembling at new division sites too early in the cell cycle. If this is true, then earlier accumulation of FtsZ at the division site in the absence of Noc and Spo0J would be expected to result in septum formation over the DNA (i.e. guillotining of the DNA) in cells actively replicating DNA. We, therefore, examined the frequency of DNA guillotined by septa in *noc*, *spo0J* and in a *noc spo0J* double mutant (Fig. 7). We used the FM‐464 dye to visualise the membrane and DAPI to visualise the DNA. Since the frequency of guillotining of DNA by septa is relatively low even in *noc* mutants (Sievers *et al.*, 2002), we exacerbated this possible phenotype by overproducing FtsZ which has been shown to result in premature Z ring assembly at division sites (Rodrigues and Harry, 2012). To this end, all strains examined contained an IPTG‐inducible copy of *ftsZ* at the *amyE* locus. As expected, in the absence of IPTG, in otherwise wild‐type cells we rarely observed guillotining of the DNA (0.4%, Fig. 7A and B). In the absence of either *spo0J* or *noc*, or both *spo0J* and *noc*, we observed a threefold increase of guillotining events, above 1% for all mutants (Fig. 7A and B). Relative to the no‐IPTG condition, the addition of IPTG to overproduce FtsZ resulted in threefold increase in guillotining events in wild‐type cells (1.1%), a 4.6‐fold increase in the *spo0J* mutant (5.9%), sixfold increase in the *noc* mutant (6.8%) and 10‐fold increase in the *spo0J noc* double mutant (10.4%). These data suggest that the role of Noc and Spo0J in ensuring that Z ring formation does not occur too early in the cell cycle, also acts to prevent significant guillotining of the DNA by the division septum.

## Discussion

In previous work, we identified a link between the initiation of DNA replication and Z ring positioning. Here, we examine the extent to which chromosome organisation might be involved in connecting these two cell cycle processes. We reveal a new role for the chromosome organisation protein, Spo0J, in preventing midcell Z ring assembly during these early stages of DNA replication. This is akin to the role of the nucleoid occlusion protein, Noc. We further show that the absence of both Noc and Spo0J when the early stages of DNA replication are blocked allows a wild‐type frequency of midcell Z rings. This indicates Noc and Spo0J function together to ensure that Z ring assembly at midcell does not occur prematurely. Consistent with this idea, we show that in cells replicating their DNA normally, Spo0J and Noc are required for the exquisite timing of Z ring assembly during the cell cycle. In other words, Noc and Spo0J, collectively ensure that Z rings don't form too early in the cell cycle. Their failure to do so results in a significant increase in nucleoids being guillotined by the division septum. The implications of our findings and how these two factors together influence Z ring positioning are discussed.

### Z ring positioning is not potentiated by the progression of initiation of DNA replication

Research over the past two decades examining how bacteria position their division site led to the idea that there is a link between the early stages of DNA replication and Z ring positioning in *B. subtilis* (Harry *et al.*, 1999; Regamey *et al.*, 2000; Moriya *et al.*, 2010). These observations culminated in the Ready‐Set‐Go model. This model proposed that the progression of initiation of DNA replication, leading up to the assembly of the replication machinery at *oriC*, coincides with an increase in potential to form a Z ring at midcell (Moriya *et al.*, 2010), with the Z ring only assembling at midcell once the chromosome is at least 70% replicated and nucleoid occlusion is relieved at that site. We proposed this model because in the absence of Noc, we observed an increase in the frequency of midcell Z rings as the early stages of DNA replication progressed. However, our new data here show that regardless of the stage of replication arrested, the absence of both *noc* and *spo0J* completely restores midcell Z ring frequency to wild‐type levels. Thus, our data are not consistent with this model. Rather our data support the idea that the midcell site is fully potentiated for Z ring assembly during the early stages of replication (at least from DnaB activity onward) and the prevention of midcell Z rings is solely reliant on Noc and Spo0J.

The question remains, what is the positional cue or signpost for midcell Z ring assembly? The idea of a signpost came from our previous work in *B. subtilis* (Rodrigues and Harry, 2012) that showed in the absence of both the Min system and Noc (and the nucleoid itself), midcell Z ring positioning is as precise as that in wild‐type cells. Given our finding here that the earliest block to initiation of DNA replication (*dna‐1*) allows for complete midcell Z ring assembly when both *spo0J* and *noc* are absent, the putative signpost for midcell Z ring assembly is likely determined prior to the loading of the DnaC helicase at the origin, an event requiring DnaB, and that Spo0J and Noc contribute to blocking the utilisation of this signpost until most of the chromosome has cleared midcell.

### How does Spo0J function in controlling midcell Z ring assembly?

A role for Noc in preventing midcell Z ring assembly over unreplicated DNA is well documented (Wu *et al.*, 2009). However, a similar role for Spo0J is less clear. In its role as a chromosome organisation protein, Spo0J binds to DNA at *parS* sites surrounding the origin, and bridges neighbouring DNA to form nucleoprotein complexes (Murray *et al.*, 2006; Graham *et al.*, 2014; Chen *et al.*, 2015). Spo0J then recruits SMC to these sites for full condensation of the chromosome (Marbouty *et al.*, 2015; Wang *et al.*, 2017). Our observations are fully consistent with recent data showing that Spo0J, by recruiting SMC, plays an important role in promoting interactions between the two arms of the chromosomes and condensation of the chromosome (Wang *et al.*, 2014b; Wang *et al.*, 2015; Marbouty *et al.*, 2015; Wang *et al.*, 2017). However, the inhibitory action of Spo0J on Z ring assembly at midcell observed here cannot be fully explained by Spo0J's role in recruiting SMC. This is because when SMC is degraded in the *dna‐1* mutant at the non‐permissive temperature, midcell Z ring assembly does not phenocopy a *spo0J* null, nor does the degradation of SMC in the *dna‐1 noc* mutant fully restore midcell Z rings to the extent of a *dna‐1 spo0J noc* double mutant.

Apart from recruiting SMC, what additional action of Spo0J is required to prevent midcell Z ring assembly during the early stages of replication? We hypothesise that the second way in which Spo0J exerts a negative effect on midcell Z ring positioning is through its ability to bind DNA and form large nucleoproteins complexes that hinder midcell Z ring assembly over the DNA, in a similar fashion to its homologue Noc (Wu *et al.*, 2009; Adams *et al.*, 2015). Importantly, disrupting the formation of Spo0J nucleoprotein complexes does not appear to affect the gross morphology of the nucleoid, at least in non‐replicating cells, since the absence of Spo0J when SMC is degraded does not alter the morphology of the nucleoid to an even less compact state (Fig. 5A). Thus, Spo0J and Noc are perhaps more similar than we think: like Spo0J, Noc binds the DNA and spreads to form nucleoprotein complexes, albeit Noc nucleoprotein complexes do not appear to exert a major effect on chromosome organisation, at least at a gross morphological level.

A recent model proposed by Errington and co‐workers suggests that, by simultaneously binding to specific DNA sites (Noc‐binding sites; NBS) and by weakly interacting with the membrane, Noc transiently tethers the DNA to the membrane periphery (Adams *et al.*, 2015). By doing so, regions of DNA/protein crowding are generated between the DNA and the membrane periphery which hinders the ability of the division machinery to assemble over the DNA (Adams *et al.*, 2015). This model for Noc function incorporates aspects of the classic nucleoid occlusion model proposed by Woldringh, which postulated that regions of transertion (coupled transcription‐translation and subsequent membrane protein insertion) could lead to steric crowding between the inner membrane and the DNA to prevent the assembly of the Z ring over the DNA (Mulder and Woldringh, 1989; Woldringh *et al.*, 1990; Woldringh *et al.*, 1991; Woldringh, 2002). In this light, Noc is a mediator of nucleoid occlusion, rather than a bona fide nucleoid occlusion protein that directly inhibits Z ring assembly over the DNA. Interestingly, Errington and colleagues showed that expression of a Spo0J‐hybrid, containing the N‐terminal, membrane binding amphipathic helix of Noc, in *E. coli* resulted in a distinct block to cell division in otherwise normally growing cells (Adams *et al.*, 2015). Although these experiments were performed in a heterologous system, this suggests that Spo0J, like Noc, can mediate a steric crowding effect on the division machinery when tethered weakly to the membrane. Based on their sequence identity and their capacity to bind and spread on the DNA, it has been hypothesised that Noc has become a repurposed nucleoid occlusion version of Spo0J (Adams *et al.*, 2015). However, considering the heterologous Spo0J‐hybrid experiments and the data we present here, it seems possible that Spo0J, like Noc, has the capacity to orchestrate a nucleoid occlusion effect and Noc evolved as a more sophisticated mediator of nucleoid occlusion.

It seems unlikely, however, that Spo0J functions like Noc in mediating nucleoid occlusion. In otherwise wild‐type cells, we observed that a *minCD spo0J* double mutant does not result in inhibition of cell division like a *noc minCD* double mutant at either 30°C or 37°C (Fig. S11; Wu and Errington, 2004). Thus, one possibility is that, in comparison to Noc, Spo0J may generate more localised areas of protein crowding around the origin‐proximal region, rather than across the bulk of the chromosome and between the chromosome and the membrane. Recent work suggests that Spo0J assembles a higher order nucleoprotein complex at the origin‐proximal region through DNA bridging that connects distant DNA loci and traps large DNA loops over multiple kilobases (Murray *et al.*, 2006; Breier and Grossman, 2007; Graham *et al.*, 2014). This origin‐proximal region, where *parS* sites are also located and Spo0J binds to, contains a large number of highly transcribed ribosomal loci that through the bridging action of Spo0J could become a chromosomal domain of high transcription and translation (Jarvis *et al.*, 1988; Davies and Lewis, 2003; Couturier and Rocha, 2006). The high concentration of RNA polymerases and ribosomes required to transcribe and translate these highly transcribed ribosomal loci would generate self‐sustaining steric crowding around the DNA that could possible emanate close to the membrane periphery. The steric crowding generated by this high concentration of large protein complexes, and associated activities, could physically occlude Z ring assembly as suggested previously by Woldringh and co‐workers (Woldringh *et al.*, 1990; Woldringh *et al.*, 1991; Woldringh, 2002). When *spo0J* is deleted in conditions where DNA replication is blocked, these affects would be dissipated partly by the loss of Spo0J nucleoprotein complexes and partly by the loss of SMC‐mediated chromosome compaction, that collectively would disperse molecular crowding around the DNA and subsequently allow Z rings to form more readily at midcell over the unreplicated, unsegregated DNA.

Thus, it appears that an emerging theme from our work and that of others, is that nucleoid occlusion may well be the result of molecular crowding forces around the DNA, and Noc and Spo0J help to organise molecular crowding in such a way that make it an effective body‐guard against division occurring over the nucleoid. The question then becomes, are these molecular crowding forces just purely a physical effect or is there is a direct genetic component that senses crowding and feeds this information back to the division apparatus? We cannot exclude the possibility that the absence of Spo0J compromises the function of an as yet unidentified nucleoid occlusion protein(s).

### Spo0J and Noc are required to fine‐tune thetiming of Z ring assembly at division sites in replicating cells

Examination of cells replicating their DNA normally showed that Noc and Spo0J are required to ensure that FtsZ does not ‘jump‐the‐gun’ in terms of its assembly at new division sites (Fig. 6). A consequence of ‘jumping‐the‐gun’ in *noc* and *spo0J* mutants is that guillotining of the nucleoid by the division septum is observed at a higher frequency (Fig. 7). This suggests that the appropriate timing of FtsZ accumulation at the new division sites prevents guillotining of the DNA when the division septum closes down later in the cell cycle. Interestingly, in the absence of *noc* and *spo0J* it appears that the nucleoids segregate slightly earlier in the cell cycle, relative to wild type. In the case of Noc, this would suggest that it somehow contributes to chromosome segregation. Given the homology of Noc to Spo0J (36% sequence identity (Sievers *et al.*, 2002)), this may not be so surprising. In fact, previous work indicates that Noc may reside in a complex with SMC (Gruber and Errington, 2009). Thus, one possibility for FtsZ accumulating earlier in the cell cycle in the absence of Spo0J and Noc, is earlier segregation of the nucleoids and consequently earlier relief of nucleoid occlusion. However, since we observed guillotining of the DNA by the division septum in* noc* and *spo0J* mutants, another possibility is that in the absence of either protein, nucleoid occlusion effects, as hypothesised above, are dissipated too early in the cell cycle. These possibilities are not mutually exclusive.

Clearly, future work is required to tease out the underlying forces of nucleoid occlusion, how they are intimately linked to chromosome organisation, and how they ensure a tight coordination between Z ring assembly and the activities of the nucleoid.

## Experimental procedures

### Bacterial strains and growth conditions

All strains, plasmids and oligonucleotides used in this study are listed in Tables S1 and S2 in the supplemental material. Cloning and genetic manipulations were carried out using standard techniques (Sambrook and Russell, 2001). Plasmid construction is detailed in the supplemental material. *B. subtilis* strains were grown vegetatively on tryptose blood agar plates, or in Penassay broth (PAB) or LB medium at 30°C. Antibiotics were used at the following concentrations: chloramphenicol,5 µg ml^−1^; erythromycin, 0.5 µg ml^−1^; neomycin, 3 µg ml^−1^; phleomycin, 0.4 µg ml^−1^; spectinomycin, 60 µg ml^−1^; tetracycline, 8 µg ml^−1^. Growth temperatures are specified in text. To induce expression of *ftsZ–yfp*, 0.02% (w/v) xylose was included in the medium. To induce the SMC‐degradation system, 1% (w/v) xylose was included in the medium.

Spores were prepared and harvested as described previously (Migocki *et al.*, 2004). Spore germination and outgrowth was performed with 2 × 10^8^ spores ml^−1^ in GMD at 34°C (Harry *et al.*, 1999; Regamey *et al.*, 2000). Antibiotic selection was not applied during spore outgrowth. HPUra was added, where indicated, to a final concentration of 100 mM at the very beginning of the incubation. The time taken for spores to germinate varies between strains and the reason why is because the time taken for outgrown cells to attain a certain length varies. However, the cell length of outgrown cells can be used as an indication of the stage of the cell cycle. For outgrowth at the non‐permissive temperature, spores were germinated 15–20 min at 34°C with shaking and then transferred to 48°C for 70–90 min, with shaking. For outgrowth at the permissive temperature, spores were incubated at 34°C with shaking for 110–140 min.

### Microscopy and image analysis

Samples were prepared for live cell fluorescence and immunofluorescence microscopy as described previously (Moriya *et al.*, 2010). For examination of nucleoid morphology in compliment of the cells prepared for immunofluorescence microscopy, cells were fixed in 70% ethanol according to the method of Hauser and Errington (Hauser and Errington, 1995). Samples were viewed using a Zeiss Axioplan 2 fluorescence microscope equipped with a 100x Plan ApoChromat phase‐contrast objective (NA 1.4; Zeiss) and an AxioCamMRm cooled charge‐coupled‐device (CCD) camera. The light source was a 100 W high‐pressure mercury lamp passed through the following filters: for visualising Alexa 488 (Filter set 09, Zeiss), for visualising DAPI (Filter set 02, Zeiss; 365), for visualising CFP (Filter Set 31044 v2, Chroma Technology), for visualising YFP (Filter set 41029, Chroma Technology) and for visualising FM4‐64 (Filter set 15, Zeiss; 546–558 nm BP excitation filter, 550 nm LP barrier filter). Images were collected using the AxioVision software program, version4.8 (Zeiss). Cell length measurements were recorded using Axiovision (Zeiss), while statistical analysis was performed in the program Excel (Microsoft). Population demographs were constructed using Oufti (Paintdakhi *et al.*, 2016). Phase contrast and FM4‐64 raw images were merged with ImageJ (Schneider *et al.*, 2012) and were used as the image in Oufti to determine individual, divided cells. YFP and DAPI signals were used to determine FtsZ and DNA localisation respectively. Background fluorescent signals were subtracted in Oufti. Cell populations were limited to 600 cells per sample to ensure equal populations across all strains.

### Measurement of Z ring and foci positioning

Cell length values were scored directly from digital micrographs using AxioVision software, version 4.8 (Zeiss), with the appropriate scaling. The positioning analysis of fluorescent signals of interest (including the Z ring seen as a band and *oriC* seen as a focus within the cell) was determined by measuring the distance from the fluorescence signal to the nearest cell pole and by dividing this value by the cell length, with 0.5 being exactly midcell. Numerical values derived for each cell length and position was exported from the AxioVision software as a text file, and imported into Excel (Microsoft) for data processing and statistical analysis. Prism7 (GraphPad) was used to generate the graphs presented in this study.

### Statistical analysis

Statistical analysis of the data obtained was carried out using the ‘Kolmogorov–Smirnov’ test (http://www.physics.csbsju.edu/stats/KStest.n.plot_form.html). The ‘Kolmogorov–Smirnov’ test was used to compare the precision of midcell Z ring or LacI‐YFP/CFP foci distances between wild‐type and mutant strains. This test was performed using a 95% confidence interval, where *P* < 0.05 was indicative of a statistically significant difference between the data sets compared.

### DNA content quantification via flow cytometry

To examine DNA content per cell, flow cytometry was used as described previously (Okumura *et al*, 2012). After the addition of chloramphenicol to the samples, cells were grown at their specific conditions for a further 5 h: *dna‐1* condition at 48°C and +HPUra condition at 34°C. SYTO16 was used to stain the DNA (1 µM; Molecular Probes). The DNA content of the cell suspensions was measured using an LSRII (Becton Dickinson), and data were collected using FACSDiva software (version 8.0.1; Becton Dickinson).

### Immunoblot analysis

Spores were germinated and outgrown as required and cell samples were equalised based on absorbance (OD_600_). Lysates were prepared as described previously (Harry*et al.*, 1993). Samples were heated for 2 min at 95°C, and centrifuged for 5 min before loading. Proteins were separated by SDS‐PAGE on a 7.5% polyacrylamide gel, electroblotted onto polyvinylidene difluoride (PVDF) membrane (BioRad) and blocked in 5% skim‐milk in phospohate‐buffered saline (PBS). The blocked membrane was probed with anti‐SMC (1:1,000) or anti‐FtsZ (1:1,000) diluted in 5% skim‐milk in PBS. Primary antibodies were detected using horseradish peroxidase‐conjugated goat anti‐rabbit IgG (Promega), the ECL chemiluminescent detection reagents (enhanced chemiluminescence; GE Healthcare) and a ChemiDoc XRS+ imaging system (Bio‐Rad).

## Conflict of interest

The authors declare that they have no conflict of interest.

## Data Availability

The data that support the findings of this study are available from the corresponding author upon reasonable request.
