# Supplementary material for: The ParB homologs, Spo0J and Noc, together prevent premature midcell Z ring assembly when the early stages of replication are blocked in Bacillus subtilis
Source: Mol Microbiol. 2019 Jun 11;112(3):766–84. doi: 10.1111/mmi.14319 (PMC6852036; doi:10.1111/mmi.14319)
Supplement: Supplementary file 1 [file MMI-112-766-s001.pdf]

The ParB homologs, Spo0J and Noc, together prevent premature midcell Z ring assembly when the early stages of replication are blocked in *Bacillus subtilis*.

SUPPLEMENTARY MATERIALS

Isabella V. Hajduk, Riti Mann, Christopher D. A. Rodrigues, Elizabeth J. Harry

[1]The ithree institute, University of Technology Sydney, PO Box 123 Broadway NSW 2007 Australia

Running title: ParB homologs block premature midcell Z ring assembly

For correspondence:

Elizabeth.Harry@uts.edu.au

The ithree institute
Faculty of Science
University of Technology Sydney
T. +61 (02) 9514 4173
M. +61 (0) 404 643 181
PO Box 123 Broadway NSW 2007 Australia

Table S1: *Bacillus subtilis* strain list used in this study

Strain	Genotype	Source
SU5	168 *trpC2*	E. Nester
SU492	168 *trpC2 amyE::(spc* P_{xyl}*-ftsZ-yfp)*	Lab stock
SU504	168 *trpC2 amyE::Pspachy-ftsZ (cat)*	Rodrigues & Harry, 2012
SU533	168 *trpC2 Δnoc::cat*	Lab stock
SU629	168 *trpC2 Δnoc::tet amyE::(spc* P_{xyl}*-noc-yfp)*	Lab stock
SU656	168 *trpC2 Δnoc::tet*	Lab stock
SU661	168 $trpC2^{+}$ *dna-1*	Rodrigues & Harry, 2012
SU682	168 *trpC2 amyE::(spc Pxyl-ftsZ-yfp) ΔminCD::cat*	Lab stock
SU746	168 $trpC2^{+}$ *dna-1 amyE::(spc* P_{xyl}*-ftsZ-yfp)*	This work
SU747	*trpC2 Δsoj::neo*	Scholefield *et al*, 2011
SU748	*trpC2 Δspo0J::neo, amyE::spo0J (cat)*	Gruber & Errington, 2009
SU765	168 *trpC2 Δsoj-spo0J::tet*	This work
SU766	168 $trpC2^{+}$ *dna-1 Δsoj-spo0J::tet*	This work
SU767	168 *trpC2 amyE::(spc* P_{xyl}*-ftsZ-yfp) Δsoj-spo0J::tet*	This work
SU768	168 $trpC2^{+}$ *dna-1 amyE::(spc* P_{xyl}*-ftsZ-yfp) Δsoj-spo0J::tet*	This work
SU769	168 *trpC2 Δspo0J::kan*	This work
SU770	168 $trpC2^{+}$ *dna-1 Δspo0J::kan*	This work
SU771	168 *trpC2 Δsoj::kan*	This work
SU772	168 $trpC2^{+}$ *dna-1 Δsoj::kan*	This work
SU802	168 $trpC2^{+}$ *dna-1 Δnoc::cat*	This work
SU803	168 *trpC2 Δnoc::cat Δsoj-spo0J::tet*	This work
SU804	168 $trpC2^{+}$ *dna-1 Δnoc::cat Δsoj-spo0J::tet*	This work
SU823	168 *trpC2 Δsoj-spo0J::tet hutM(345°)::lacO(cat), thrC (283°)::lacI-cfp(erm) amyE::(spc* P_{xyl}*-ftsZ-yfp)*	This work
SU824	168 $trpC2^{+}$ *dna-1 Δsoj-spo0J::tet hutM(345°)::lacO(cat), thrC (283°)::lacI-cfp(erm) amyE::(spc* P_{xyl}*-ftsZ-yfp)*	This work
SU827	168 *trpC2 Δnoc::cat Δsoj::kan*	This work
SU828	168 $trpC2^{+}$ *dna-1 Δnoc::cat Δsoj::kan*	This work
SU829	168 *trpC2 Δnoc::cat Δspo0J::kan*	This work
SU830	168 $trpC2^{+}$ *dna-1 Δnoc::cat Δspo0J::kan*	This work
SU831	168 *trpC2 Δnoc::cat amyE::(spc* P_{xyl}*-noc-yfp)*	This work
SU832	168 $trpC2^{+}$ *dna-1 Δnoc::cat amyE::(spc* P_{xyl}*-noc-yfp)*	This work
SU833	168 *trpC2 Δnoc::cat Δspo0J::kan amyE::(spc* P_{xyl}*-noc-yfp)*	This work
SU834	168 $trpC2^{+}$ *dna-1 Δnoc::cat Δspo0J::kan amyE::(spc* P_{xyl}*-noc-yfp)*	This work
SU835	168 *trpC2 Δnoc::cat Δsoj-spo0J::tet amyE::(spc* P_{xyl}*-ftsZ-yfp)*	This work
SU836	168 $trpC2^{+}$ *dna-1 Δnoc::cat Δsoj-spo0J::tet amyE::(spc* P_{xyl}*-ftsZ-yfp)*	This work
SU849	*smc-ssrA loxP (kan), lacA::*P_{xyl}*A Ec sspB loxP (erm)*	Wang *et al.* 2014a
SU850	168 *trpC2 smc-ssrA loxP (kan), lacA::PxylA Ec sspB loxP (erm)*	This work
SU851	168 $trpC2^{+}$ *dna-1 smc-ssrA loxP (kan), lacA::PxylA Ec sspB loxP (erm)*	This work
SU874	168 *trpC2 Δnoc::cat, smc-ssrA loxP (kan), lacA::PxylA Ec sspB loxP (erm)*	This work
SU875	168 $trpC2^{+}$ *dna-1 Δnoc::cat, smc-ssrA loxP (kan), lacA::PxylA Ec sspB loxP (erm)*	This work
SU876	168 *trpC2 Δsoj-spo0J::tet, smc-ssrA loxP (kan), lacA::PxylA Ec sspB loxP (erm)*	This work
SU877	168 $trpC2^{+}$ *dna-1 Δsoj-spo0J::tet, smc-ssrA loxP (kan), lacA::PxylA Ec sspB loxP (erm)*	This work
SU878	168 *trpC2 Δnoc::cat, Δsoj-spo0J::tet, smc-ssrA loxP (kan), lacA::PxylA Ec sspB loxP (erm)*	This work
SU879	168 $trpC2^{+}$ *dna-1 Δnoc::cat, Δsoj-spo0J::tet, smc-ssrA loxP (kan), lacA::PxylA Ec sspB loxP (erm)*	This work
SU887	168 *trpC2 amyE::Pspachy-ftsZ (cat) Δspo0J::kan*	This work
SU888	168 *trpC2 amyE::Pspachy-ftsZ (cat) Δnoc::tet*	This work
SU889	168 *trpC2 amyE::Pspachy-ftsZ (cat) Δspo0J::kan Δnoc::tet*	This work
SU890	168 *trpC2 amyE::(spc Pxyl-ftsZ-yfp) Δspo0J::kan*	This work
SU891	168 *trpC2 amyE::(spc Pxyl-ftsZ-yfp) Δnoc::cat*	This work
SU892	168 *trpC2 amyE::(spc Pxyl-ftsZ-yfp) Δspo0J::kan Δnoc::cat*	This work
SU893	168 *trpC2 amyE::(spc Pxyl-ftsZ-yfp) Δspo0J::kan ΔminCD::cat*	This work
SU894	*yuxG(-87°)::lacO48 (phleo), yhdG(+87°)::tetO48 (cat), ycgO::*P_{ftsW} *tetR-cfp (spec) terminators* P_{ftsW} *lacI-mypet*	Wang *et al.* 2014a
SU895	*168 trpC2+ dna-1 yuxG(-87°)::lacO48 (phleo), yhdG(+87°)::tetO48 (cat), ycgO::*P_{ftsW} *tetR-cfp (spec) terminators* P_{ftsW} *lacI-mypet*	This work
SU897	*168 trpC2+ dna-1 soj-spo0J::tet, yuxG(-87°)::lacO48 (phleo), yhdG(+87°)::tetO48 (cat), ycgO::*P_{ftsW} *tetR-cfp (spec) terminators* P_{ftsW} *lacI-mypet*	This work
SU899	168 $trpC2^{+}$ *dna-1 Δspo0J::kan, ycgo::spo0J (erm)*	This work
SU901	168 $trpC2^{+}$ *dna-1 Δnoc::cat Δspo0J::kan, ycgo::spo0J (erm)*	This work
SU903	168 $trpC2^{+}$ *dna-1 Δsoj-spo0J::tet, ycgo::spo0J (erm)*	This work
SU905	168 $trpC2^{+}$ *dna-1 Δnoc::cat Δsoj-spo0J::tet, ycgo::spo0J (erm)*	This work

Table S2: Plasmids and oligonucleotides used in this study

Plasmid	Description	Source
pKM084	*ycgO::cat*	D. Rudner
pIH003	*ycgO::Psoj-optRBS-spo0J (erm)*	This work
**Oligonucleotides**	**Sequence[a]**	**Source**
oIH004	cgc**GAATTC**AAACCATTTTCTCACCATCCTG	This work
oIH005	cgc**GCTAGC**CTTTCACATGAACATGTACTATC	This work
oIH008	cgc**GCTAGC**ACATAAGGAGGAACTACTATGGCtaaAGGCCTTGGAAAAGGGAT	This work
oIH0010	gcg**GGATCC**TTATGATTCTCGTTCAGACA	This work

a Bold letters indicate the recognition sequence for restriction enzymes

Plasmid construction

pIH003 [*ycgO::Psoj-optRBS-spo0J (erm)*] was generated by a two-way ligation of a EcoRI-NheI PCR product containing the promoter region for the *soj-spo0J* operon (amplified with oligonucleotide primers oIH004 and oIH005), and a NheI-BamHI PCR product containing the *spo0J* gene (amplified with oligonucleotide primers oIH008 and oIH0010) into pKM084 cut with EcoRI-BamHI. Both PCR products were amplified from SU5 genomic DNA as the template. pKM084 is an ectopic integration vector for recombination into the *ycgO* locus (David Rudner).

SUPPLEMENTARY FIGURE LEGENDS

Figure S1: Z ring positioning when initiation of DNA replication is blocked in vegetatively grown fixed cells. (A-D) Vegetative cells were grown in PAB to the mid-exponential phase at the permissive temperature (34°C), then shifted to the non-permissive temperature (48°C) for a further 60 min: (A) wild-type; SU5, (B) *dna-1*; SU661, (C) *Δsoj-spo0J*; SU765 and (D) *dna-1 Δsoj-spo0J*; SU766. Percentages shown are the frequencies of Z rings occurring at midcell in the range of 0.45 – 0.5 on the x-axis.

Figure S2: Z ring positioning when initiation of DNA replication is blocked in vegetatively grown fixed cells of individual *soj* or *spo0J* mutants. (A-F) Vegetative cells were grown in PAB to the mid-exponential phase at the permissive temperature (34°C), then shifted to the non-permissive temperature (48°C) for a further 60 min: (i) wild-type; SU5, (ii) *dna-1*; SU661, (iii) *Δsoj*; SU769, (iv) *dna-1 Δsoj*; SU770, (v) *Δspo0J*; SU771 and, (vi) *dna-1 Δspo0J*; SU772. Percentages shown are the frequencies of Z rings occurring at midcell in the range of 0.45 – 0.5 on the x-axis.

Figure S3: Z ring positioning in outgrown spore cells at the permissive temperature. Z ring positioning was examined in outgrown spores at the permissive temperature (34°C) for 120 min in strains: (A) wild-type; SU492, (B) *dna-1;* SU746, (C) *Δsoj-spo0J*; SU767 and (D) *dna-1 Δsoj-spo0J*; SU768*.* Percentages shown are the frequencies of Z rings occurring at midcell in the range of 0.45 – 0.5 on the x-axis.

Figure S4: Whole field of view of Z rings overlayed with DAPI and phase contrast when initiation of DNA replication is blocked in live outgrown spores. Microscopic images of an overlay of phase contrast, DAPI (red) and FtsZ-YFP (green) in (A) *dna-1*; SU746, and (B) *dna-1 Δsoj-spo0J*; SU768*.* Right side images show closer details of selected cells with different nucleoid morphologies and varying Z ring positions. Scale bar represents 2 μm.

Figure S5: Flow cytometry profiles of *Δsoj-spo0J* strains when initiation of DNA replication is blocked via *dna-1* mutant or addition of HPUra. Flow cytometry profiles in controls where initiation of DNA replication is progressing normally (wild-type; SU492 and *Δsoj-spo0J*; SU767) or blocked (controls: *dna-1*; SU746 and wild-type +HPUra; and test strains: *dna-1 Δsoj-spo0J*; SU768 and *Δsoj-spo0J* +HPUra. n = 10,000.

Figure S6: Noc localisation in the absence of s*po0J* when initiation of DNA replication is blocked. Strains (A) wild type; SU831, (B) *dna-1*; SU832, (C) *Δspo0J Δnoc*; SU833, and (D) *dna-1 Δspo0J Δnoc*; SU834, possessing Noc tagged with a yellow fluorescent protein (*noc-yfp*; falsely coloured cyan) were grown vegetatively supplemented with 0.3% xylose. DNA stained with DAPI (falsely coloured red). Scale bar = 2 μm.

Figure S7: Z ring positioning when initiation of DNA replication is blocked during spore outgrowth. Z ring positioning was examined in two conditions: in the temperature-sensitive *dna-1* background (A) and with the addition of the DNA polymerase III inhibitor HPUra (B), in spore outgrown spore cells. A: (i) wild-type; SU492, (ii) *dna-1;* SU746, (iii) *Δnoc;* SU831, (iv) *dna-1 Δnoc;* SU832, (v) *Δsoj-spo0J Δnoc;* SU835, and (vi) *dna-1 Δsoj-spo0J Δnoc;* SU836; B: (i) wild-type +HPUra and (ii) *Δsoj-spo0J Δnoc* +HPUra.

Figure S8: Z ring positioning in the individual mutants, *soj* or s*po0J*, in the *dna-1 Δnoc* mutant at the non-permissive temperature. Scatter plots showing Z ring

positioning and average cell length of vegetatively grown, fixed cells of (A) wild-type; SU5, (B) *dna-1*; SU661, (C) *Δsoj Δnoc*; SU829, (D) *dna-1 Δsoj Δnoc*; SU830, (E) *Δspo0J Δnoc*; SU827, and (F) *dna-1 Δspo0J Δnoc*; SU828.

Fig S9. Complementation of spo0J in the temperature sensitive strains. (A) Z ring positioning, and (B) nucleoid morphologies in strains (i) *dna-1 spo0J ycgo::spo0J* (SU899), (ii) *dna-1 spo0J noc ycgo::spo0J* (SU901), (iii) *dna-1 soj-spo0J ycgo::spo0J* (SU903), and (iv) *dna-1 soj-spo0J noc ycgo::spo0J* (SU905), grown vegetatively at the non-permissive temperature and examined via IFM and ethanol fixation, respectively. n = 200.

Figure S10: Z ring positioning when initiation of DNA replication is blocked during spore outgrowth in the *dna-1* temperature-sensitive SMC-depleted mutant. Scatter plots showing Z ring positioning and average cell length of outgrown spores grown in the presence of xylose (1% v/v) to induce SMC degradation in replicating cells (left column) and non-replicating cells (*dna-1* mutant; right column). Z ring positioning was examined in strains (A) wild-type; SU850, (B) *dna-1;* SU851, (C) *Δnoc;* SU874, (D) *dna-1 Δnoc*; SU875, (E) *Δsoj-spo0J;* SU876, (F) *dna-1 Δsoj-spo0J;* SU877, (G) *Δnoc Δsoj-spo0J;* SU878, and (H) *dna-1 Δnoc Δsoj-spo0J;* SU879.

Figure S11: Cell division is not inhibited in a *minCD spo0J* double mutant. Phase contrast images of mid-exponentially growing cells of strains (A) wild-type; SU492, (B) *Δspo0J*; SU890, (C) *ΔminCD*; SU682 and (D) *Δspo0J ΔminCD*; SU893 grown at 30°C and 37°C. Scale bar represents 2 μm.

Hajduk et al.
Figure S1

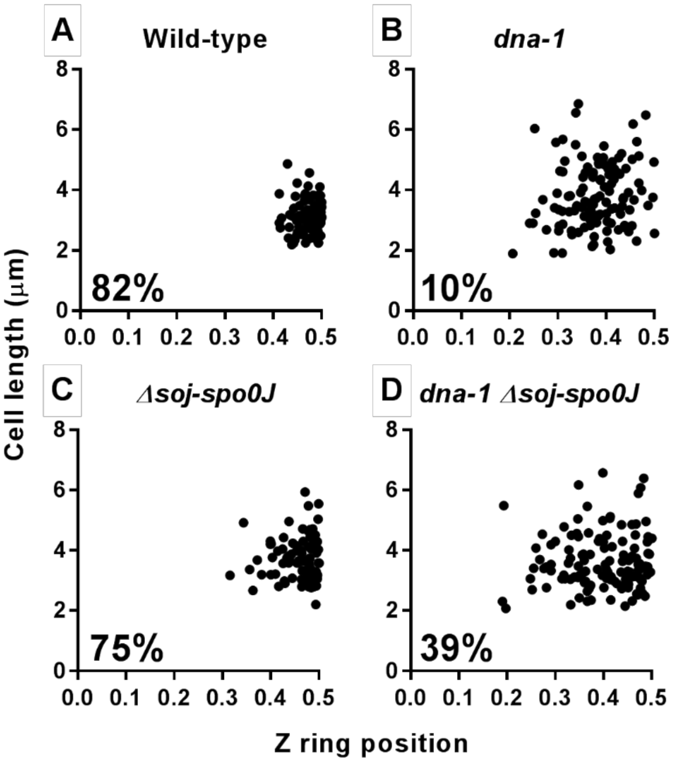

Hajduk et al.
Figure S2

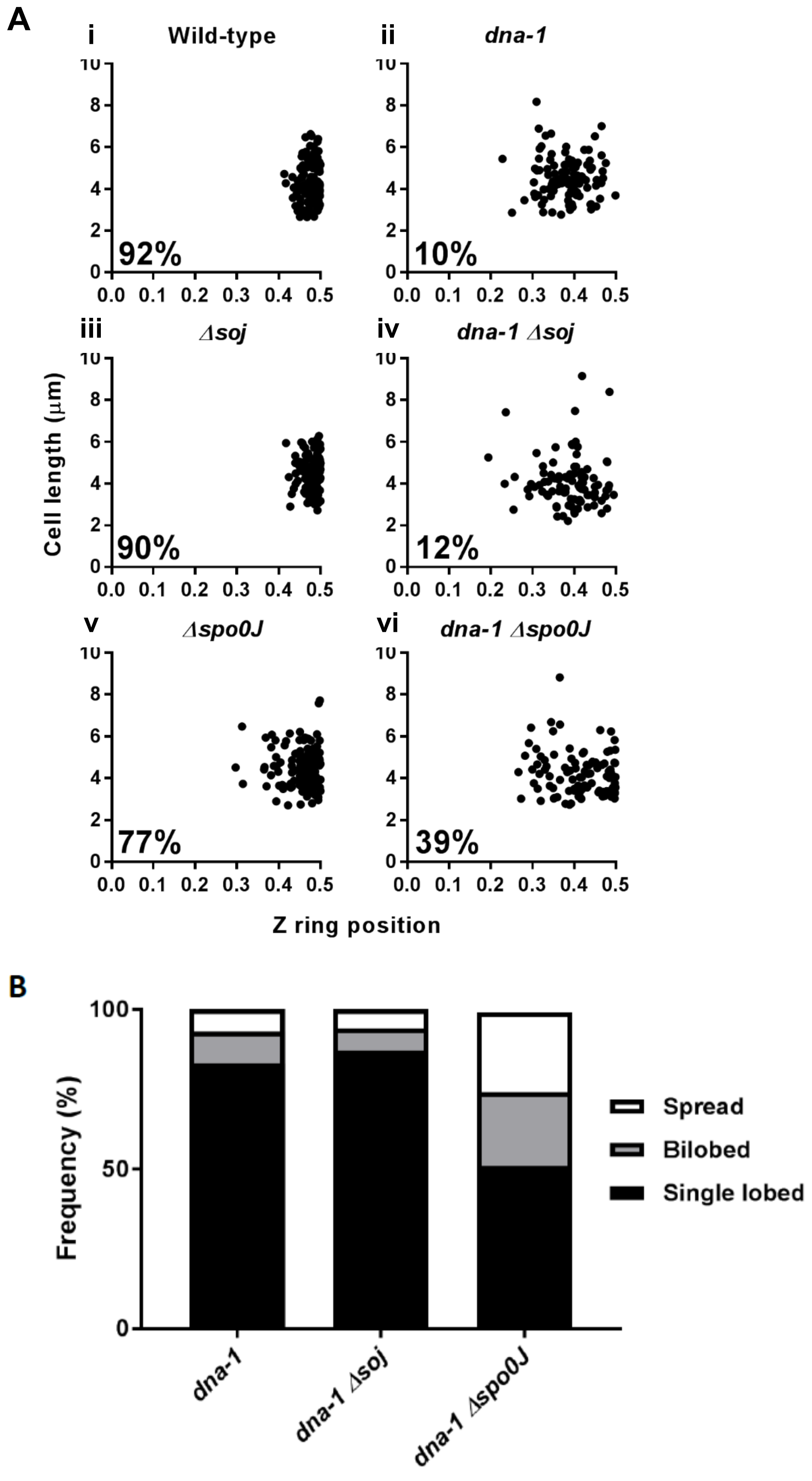

Hajduk et al.
Figure S3

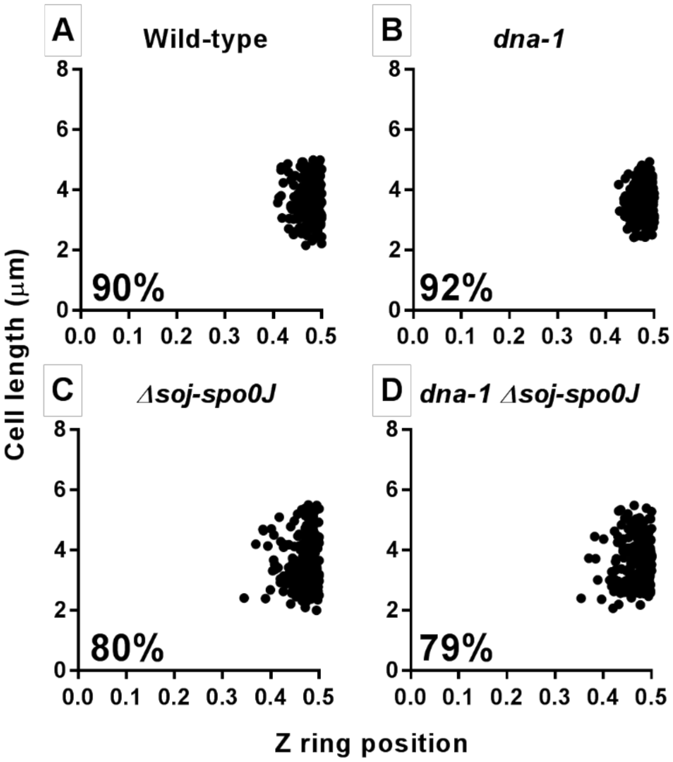

Hajduk et al.
Figure S4

A ***dna-1***

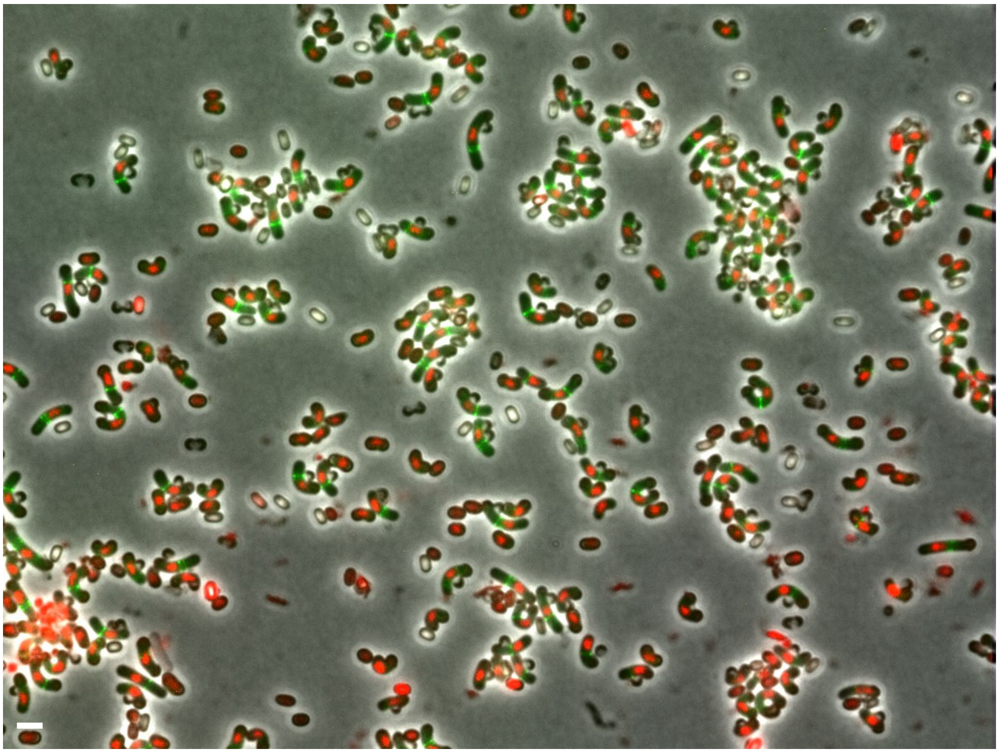

B ***dna-1 Δsoj-spo0J***

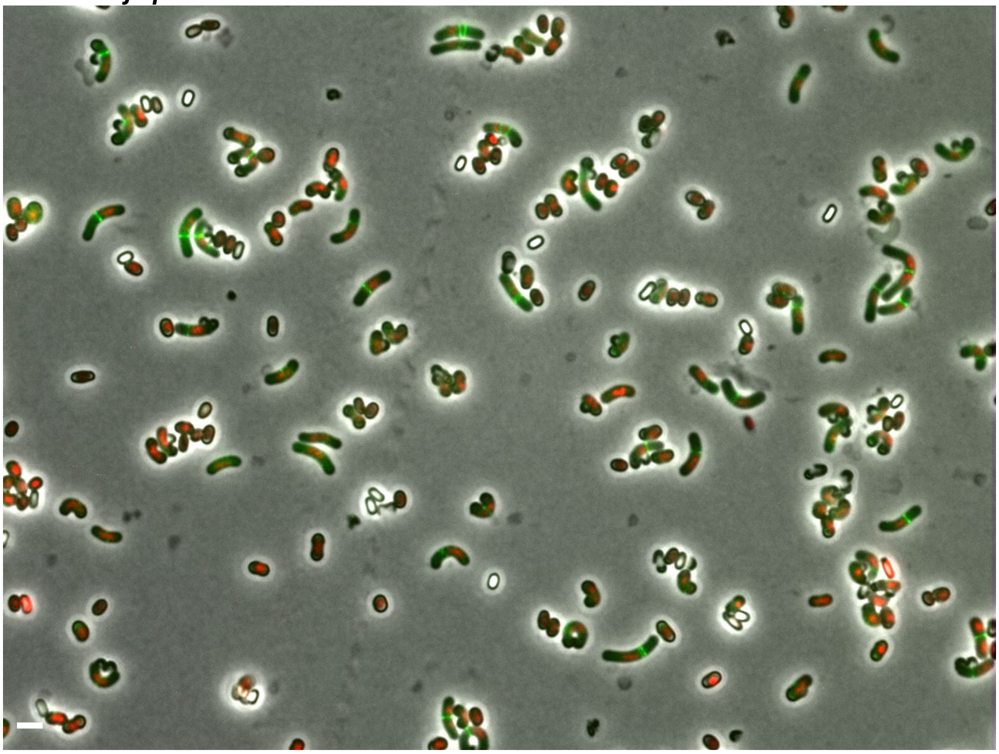

Hajduk et al.
Figure S5

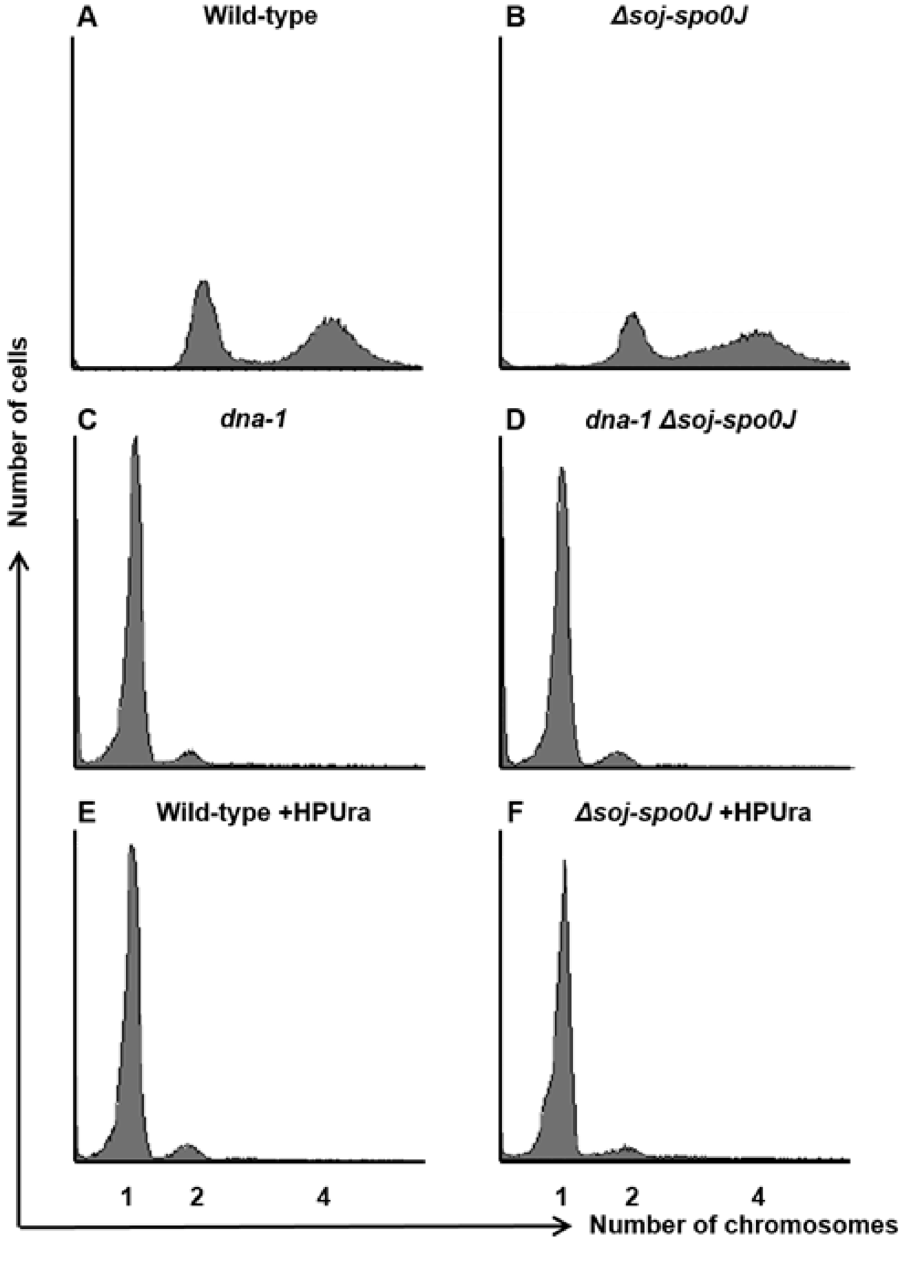

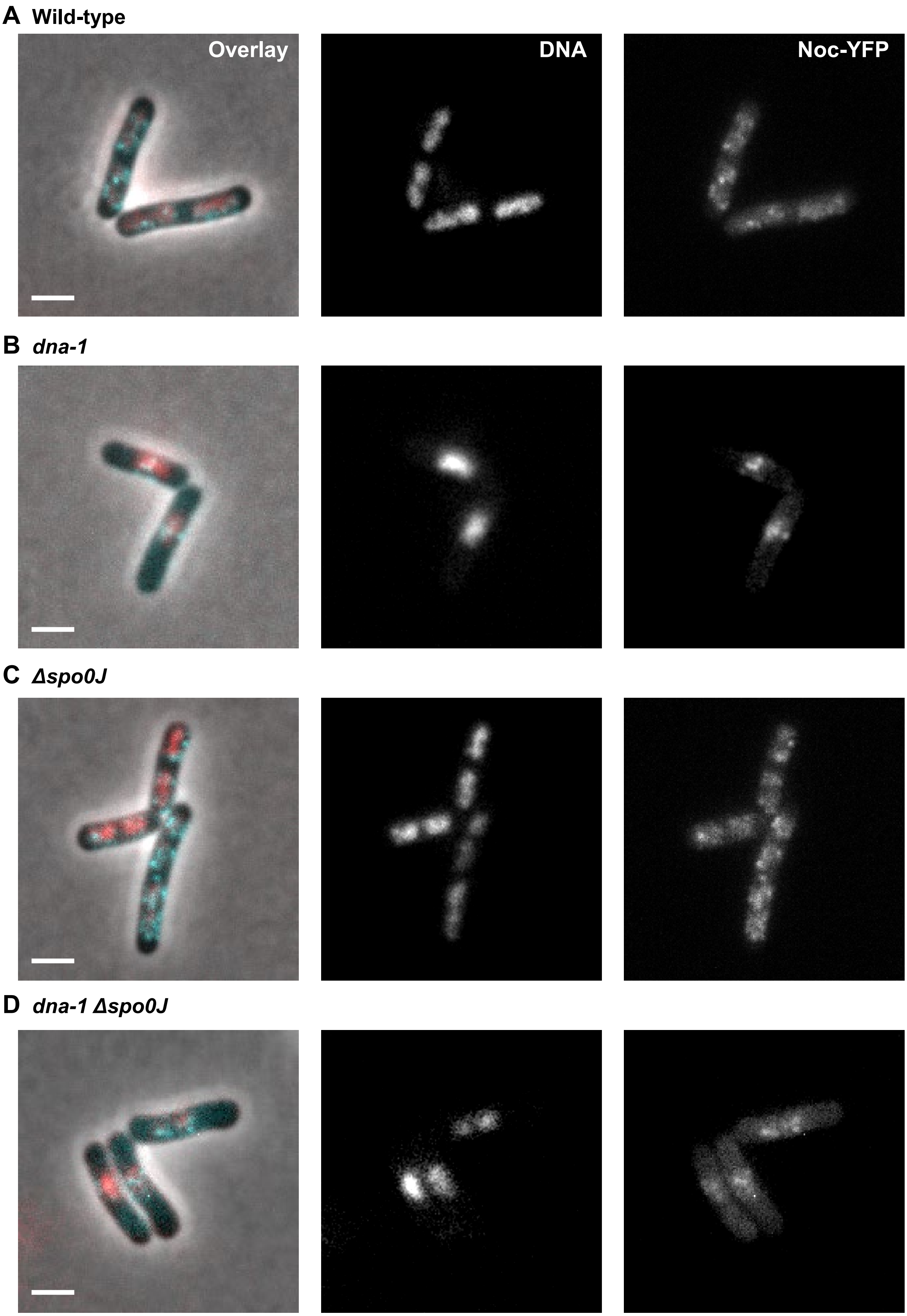
A Wild-type
Overlay
DNA
Noc-YFP
B dna-1
C Δspo0J
D dna-1 Δspo0J

Hajduk et al.
Figure S7

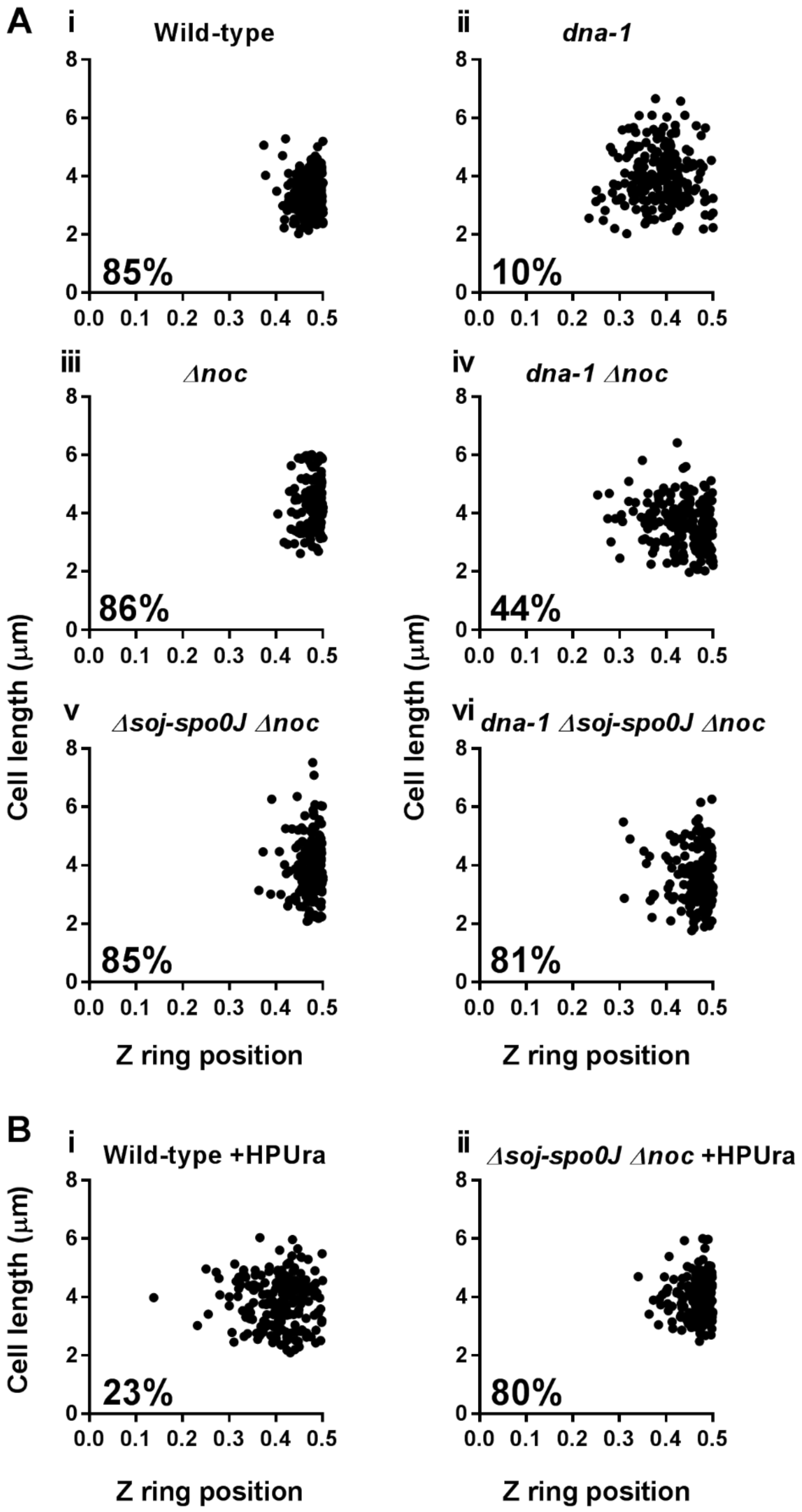

Hajduk et al.
Figure S8

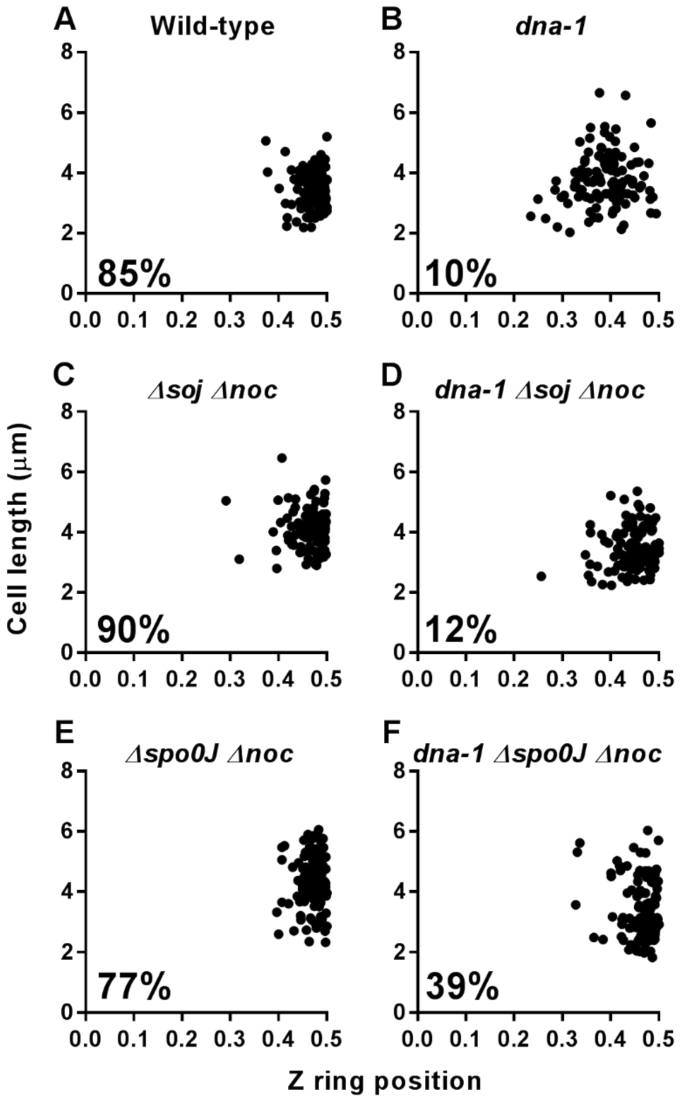

Hajduk et al.
Figure S9

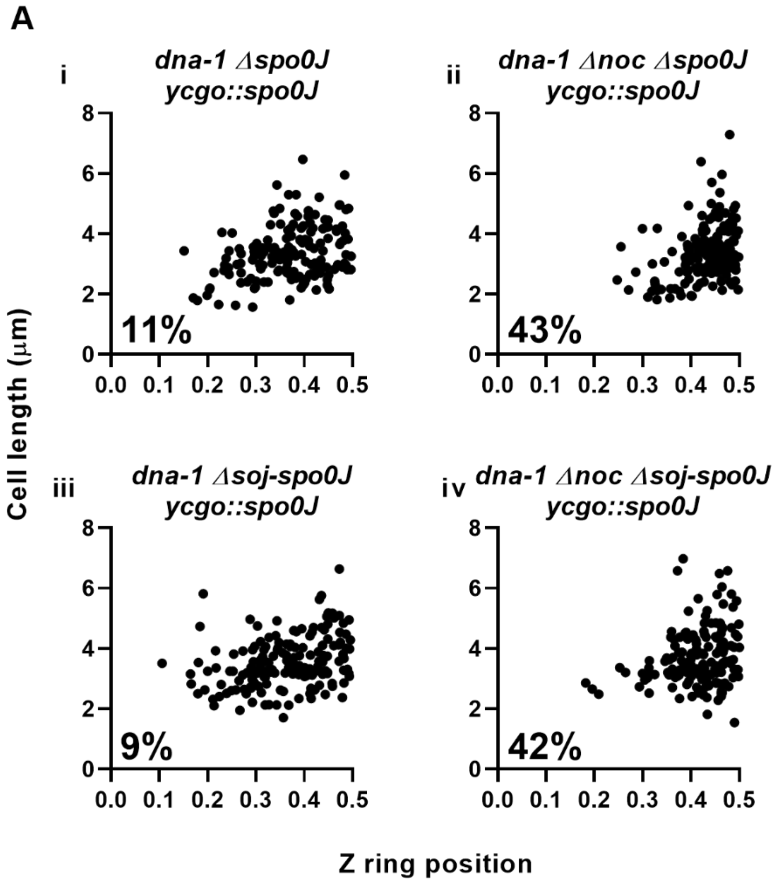

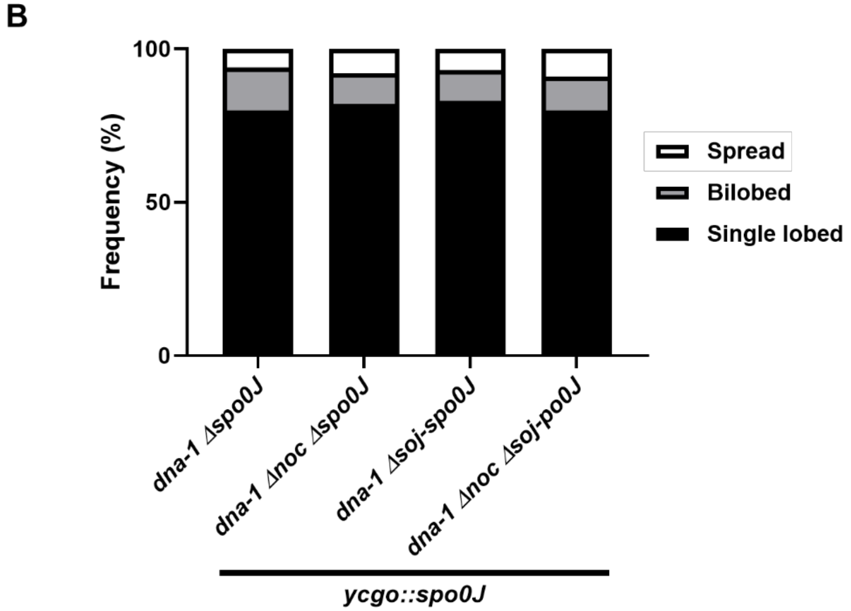

Hajduk et al.
Figure S10

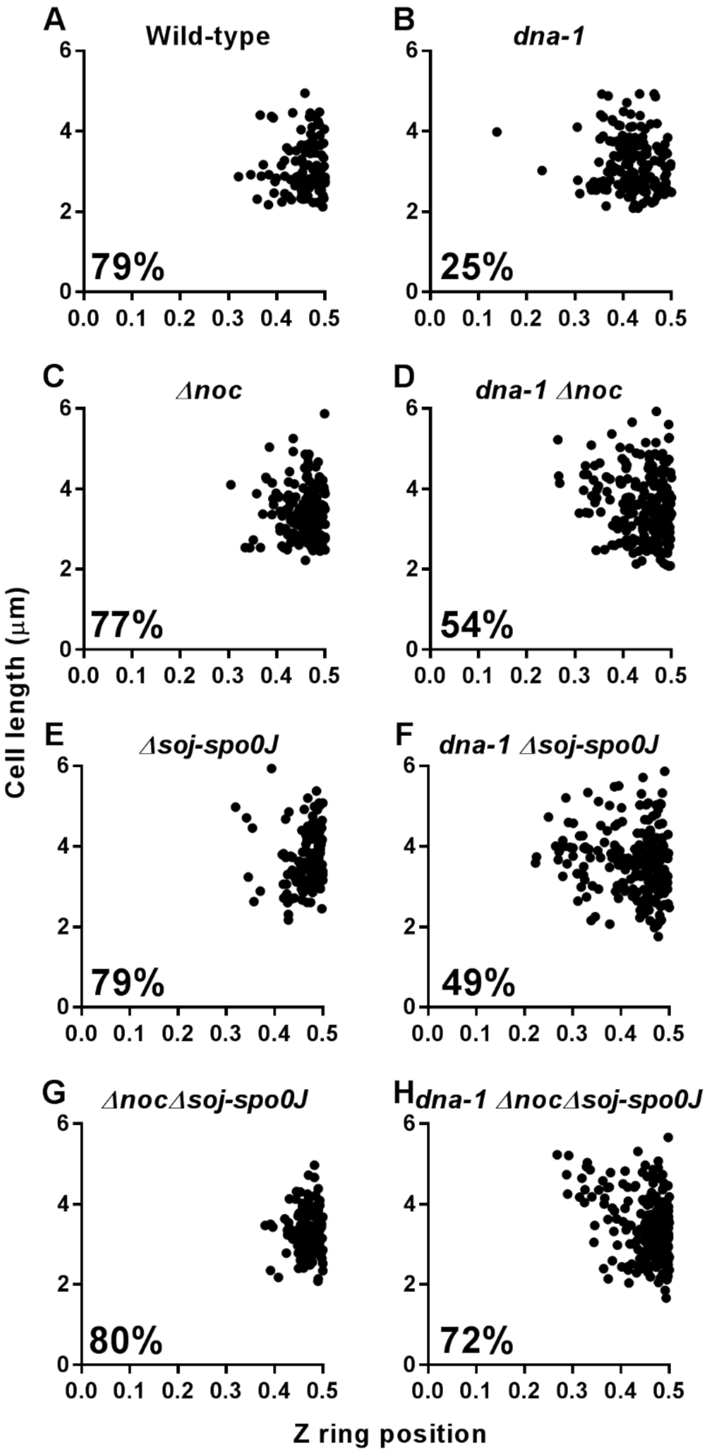

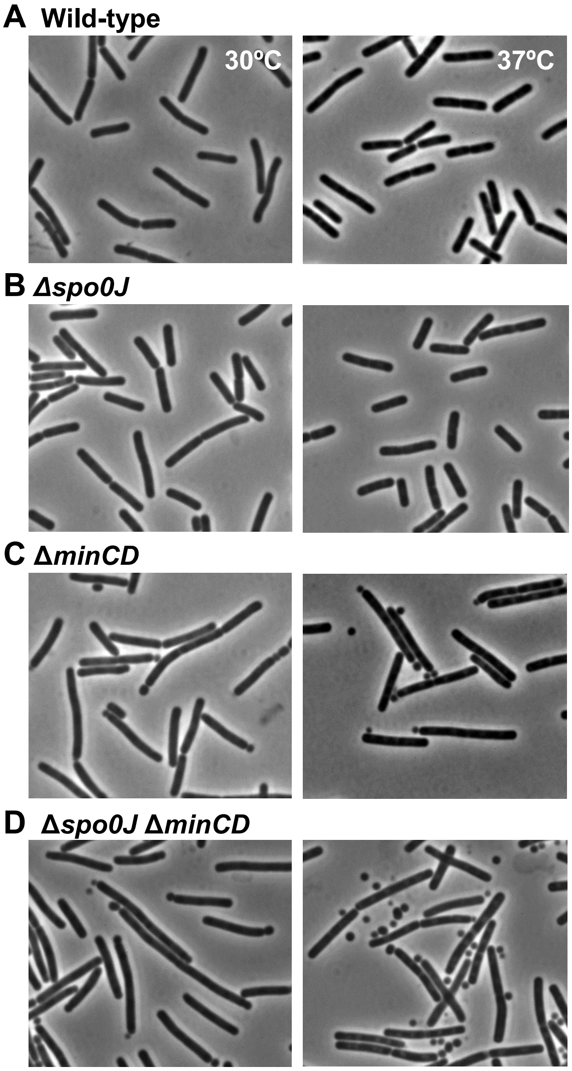
A Wild-type
30ºC
37ºC
B Δspo0J
C ΔminCD
D Δspo0J ΔminCD
